# An effective, secure and efficient tagging method for integrity protection of outsourced data in a public cloud storage

**DOI:** 10.1371/journal.pone.0241236

**Published:** 2020-11-05

**Authors:** Reem ALmarwani, Ning Zhang, James Garside

**Affiliations:** 1 College of Computer Science and Engineering (CCSE), Taibah University, Medina, Saudi Arabia; 2 Information Management Research Group, The Department of Computer Science, The University of Manchester, Manchester, United Kingdom; University College of Engineering Tindivanam, INDIA

## Abstract

Data Integrity Auditing (DIA) is a security service for checking the integrity of data stored in a PCS (Public Cloud Storage), a third-party based storage service. A DIA service is provided by using integrity tags (hereafter referred to tags). This paper proposes a novel tagging method, called Tagging of Outsourced Data (TOD), for generating and verifying tags of files. TOD has a number of unique properties: (i) it supports both public and private verifiability, and achieves this property with a low level of overhead at the user end, making it particularly attractive to mobile users with resource-constrained devices, (ii) it protects data confidentiality, supports dynamic tags and is resilient against tag forgery and tag tampering (i.e. by authorised insiders) at the same time in more secure and efficient, making the method more suited to the PCS environment, (iii) it supports tags deduplication, making it more efficient, particularly for the user who has many files with data redundancy. Comprehensive security analysis and performance evaluation have been conducted to demonstrate the efficacy and efficiency of the approach taken in the design.

## 1 Introduction

Public Cloud Storage (PCS) is one of the commonly used Cloud Computing services. Outsourcing data in a PCS can bring benefits to PCS users. Data managed by a PCS provider can be accessed anywhere, anytime and with any device, significantly increasing the accessibility and availability of data. Furthermore, the storage capacities can easily be scaled up and down based on the size of storage space subscribed by the PCS users, making the storage service provisioning more scalable and cost-effective. However, as data in PCS are managed by the PCS provider which is a third party, there are additional security concerns, and one of these concerns is how to ensure the integrity of data managed by the PCS provider.

Outsourced data can be vulnerable to accidental and intentional alterations, and these alterations may be performed by external entities as well as authorised insiders, e.g. an employee working for the PCS provider. To check the integrity of data in such an environment, a Data Integrity Auditing (DIA) service is typically used. Two integrity checking techniques have been proposed to use in the DIA, i.e., Proof of Retrievability (POR) [1] and Provable Data Possession (PDP) [2], without downloading the whole data from the PCS. These are based on spot-checking. As we are interested in dynamic data, and the PDP can support dynamic data integrity verification more efficiently; thus the paper only focuses on the PDP-based DIA solutions (use DIA to indicate PDP-based solutions).

With DIA, a PCS user generates tags for their data before uploading the data along with the tags on to a PCS server. The tags serve as the authenticators for the data, protecting its integrity. Whenever the integrity of the data is to be verified, some computations are performed on the data, and the result of the computation is compared with the associated tags. If the data have been altered, the verification will produce a negative result. Tags are generated and verified using a tagging method. For DIA to be effective, secure and efficient, we need an effective, secure and efficient tagging method.

A tagging method typically consists of two algorithms, one for tag generation (tag generation algorithm) and the other for tag verification (tag verification algorithm). Usually, a tag generation operation is performed by a PCS user (a data owner) using a tag generation algorithm to generate tags for her/his data. A tag verification operation, on the other hand, may either be performed by the data owner him/herself, in which case, the tagging method is said to support private verifiability, or by a trusted third party (Third Party Auditor (TPA)) delegated by the data owner, in which case, it is said to support public verifiability.

Over the past few years, a number of tagging methods have been proposed in the literature [3–14] to used in DIA, some [3–7] supporting private verifiability, while others [8–14] supporting public verifiability. The main focus of the existing work is on how to support public verifiability and/or to make the methods more secure or more efficient.

While existing work has made some major contributions to knowledge, there are three aspects in which the work can be further improved. The first is that existing tagging methods are largely designed to counter threats from external entities. They assume that third parties (PCS provider and/or TPA) are trustworthy. With some of the methods, the verification of the integrity of data even requires that a designated third party access plaintext data. This places unconditional trust on the third party. Should the third party misbehave, the confidentiality of data or the privacy of data owners may be put at risks. The second is that existing methods, to address the tag collision and/or data confidentiality, are not designed to support dynamic data efficiently. When modifications are made to any single data block, e.g. when a new data block is inserted, or an obsolete block is deleted, multiple tags (not just the tag associated to the affected data block) are affected and need to be re-computed. The third is that existing methods do not differentiate identical data from non-identical data, and they generate tags for identical data in the same way as for non-identical data. As a result, they generate duplicated tags for identical data, resulting in unnecessary overheads. The data deduplication is applying at the file-level, not block-level, and even that the duplication is detected, the PCS user still should generate tags. On other words, the deduplication property is not considered in designing the tagging method.

By supporting both public and private verifiability on the same platform, we can get rid of the assumption that the third parties are trustworthy. By supporting the function of public verifiability, users can delegate the tasks of data integrity verification to a third party, say TPA, to reduce overhead costs imposed on the users. By also supporting the function of private verifiability, users will have the option of performing the verifications themselves anytime to monitor the integrity of the third parties which manage their data (PCS provider and/or TPA). In this way, we can reduce overhead costs on users while at the same time also reduce trusts on third parties.

By ‘secure’, we mean that, while providing the integrity protection function, the method should also ensure the protection of data confidentiality and be resilient to attacks on tags. By ‘efficient’, we mean that the overhead costs, in term of computational cost, incurred in tag generation and verification, should be as low as possible, particularly for the user end, thus making the DIA service also suited to users with resource-constrained devices.

TOD achieves the above properties by making a hybrid use of cryptographic primitives, namely homomorphic encryption, algebraic signature and BLS short signature, as well as the ideas of tag deduplication and decoupling block indices from tag generations. TOD has been analysed and evaluated in terms of security and performance, and the results of the analysis and evaluation have been compared with related methods, demonstrating that our method is more secure and more efficient while supporting a richer set of functionality. Accurately, our contributions can be summarised as the following:

- Analyse threats to data integrity verification in a DIA system and specify a set of requirements for the design of an effective, secure and efficient tagging method.
- Analyse the existing tagging methods critically against the requirements to identify their strengths and weakness.
- Design a novel tagging method, i.e. TOD method.
- Prove the correctness of the TOD, and it can satisfy the security requirements through theoretical analysis.
- Justify the performance of TOD through theoretical and experimental analysis and comparisons with the related works.

The rest of the paper is structured as follows. Section 2 analyses security threats in relation to data integrity, and, based on the threat analysis, the section presents a set of requirements for an effective, secure and efficient tagging method. Based on the requirements, section 3 critically analyses related methods published in the literature, highlighting their limitations and the need for further work. Section 4 presents the TOD method addressing the limitations. The security analysis and performance evaluation of the method are given in Section 5 and Section 6, respectively. Finally, Section 7 concludes the paper.

## 2 Threat analysis and requirement specification

This section first analyses insider threats to data integrity verification in a DIA system. It then specifies a set of requirements for the design of an effective, secure and efficient tagging method.

### 2.1 Insider threat analysis

**Fig 1:**
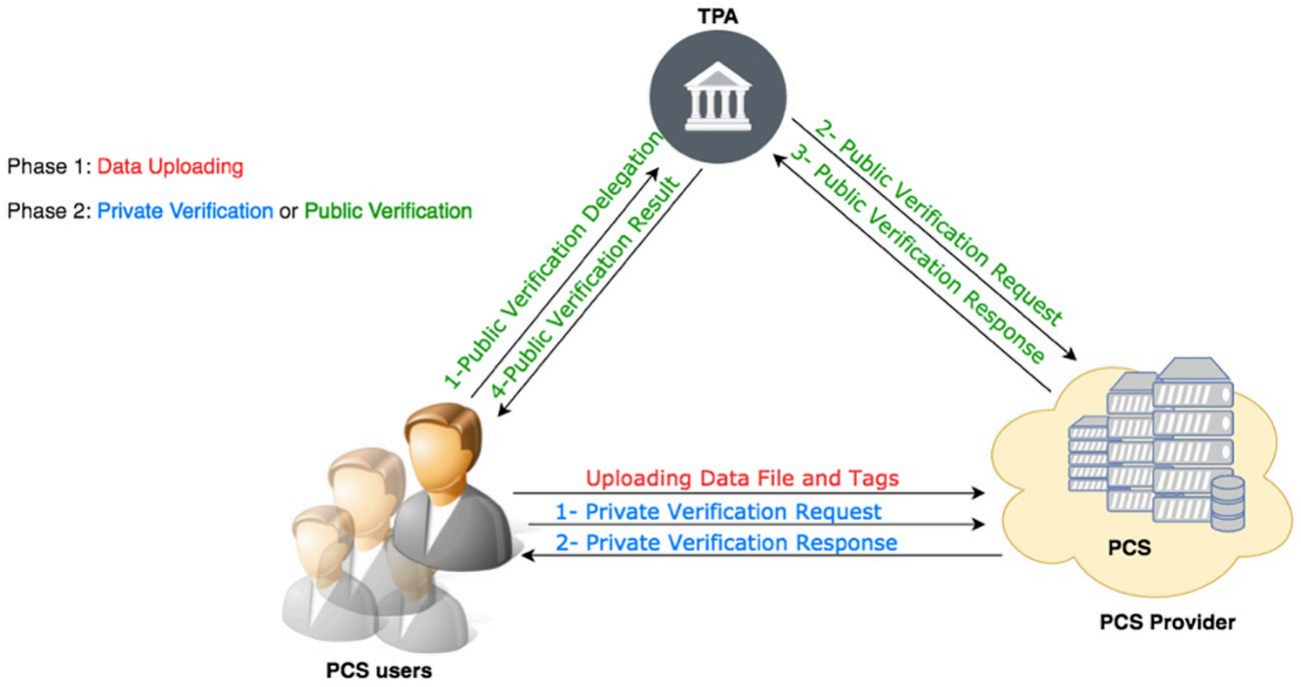
The DIA system model.

Fig 1 shows a typical DIA system model. From the figure, it can be seen that the model consists of the following entities: multiple PCS users, a PCS provider and a TPA. A PCS user is usually the owner of a data file and is responsible for generating tags for his/her data file and uploading the data file along with the tags onto the PCS. The PCS provider manages data and their associated tags for PCS users, as part of the PCS service provided to the PCS users. A verifier is an entity that verifies the integrity of the data managed by the PCS provider. A verifier can be a PCS user, i.e. the owner of the data to be verified, or a TPA. A verification operation performed by a verifier involves accessing the data and their associated tags, both of which are managed by the PCS provider, and verifying whether the tags can authenticate the given data.

In this system model, threats to data integrity can come from external entities as well as internal entities. Threats imposed by internal entities are more difficult to counter, as they are authorised insiders and have privileges to manage or access data and/or verify the integrity of data. Three types of insider threats that are related to DIA which are data integrity verification fraud, unauthorised data disclosure, and repudiation of tag generations or data updating.

- Data Integrity Verification Fraud: Data integrity verification fraud occurs when an attempt is made to cover the fact that certain PCS users’ data integrity have been compromised, such as there is loss of data or it has been altered or tampered in an unauthorised manner. It is possible that the PCS provider will manipulate or forge tags that are utilised for generating proof so that they can hide these attacks to protect their reputation. The PCS provider can do this through using three possible methods. The first is a forgery attack where tag(s) related to the data requested for a data integrity verification process is forged to ensure that the data integrity verification delivers a positive result despite the tag that is used differs from the tag that the data owner generates. For example, the PCS provider can achieve this by manipulating flaws present in a tagging method. The second is implementing a replace attack where the PCS provider utilises a tag that is generated for a piece of data different from that which is requested for verification. This replace attack is executed by using collisions between tags that are developed for data that the same user or different users own. If, for example, two PCS users have the same data blocks as well as the same tags generated for these data blocks, the PCS provider can implement the tag as well as the related data block owned by a PCS user to verify data owned by a different PCS user. Moreover, this attack can also work in case of two different data blocks having the same tags. The third way is implementing a replay attack in which the PCS provider can cache the proofs of certain data as well as tags. Upon receiving a verification request, the PCS provider dispatches the values of proofs that is cached instead of freshly generated proofs through stored data and tags within the file storage.
- Unauthorised Data Disclosure: If the user data that a PCS provider manages is not thoroughly protected, it can be disclosed because of data integrity verification operations. Both the PCS provider and the TPA have the authority to manage as well as verify the data integrity. They must not be able access the data content. If, however, they are able to access the data because of the integrity verification process, the data confidentiality and/or the data owner’s privacy can be at risks. For example, the TPA or the PCS provider’s internal employee can gain access to user data and sell it to other individuals or organisations or use it for unauthorised purposes that can hurt the users.
- Repudiation of Tag Generation: A PCS user can repudiate (falsely deny) the creation of tags for certain data to discredit or obtain certain financial gains from the TPA and/or the PCS provider. However, their denial of the tag generation can also be sincere because a PCS user (e.g., user A) could be attempting to learn another PCS user’s (user B) data by offering proof that certain tags were generated by user A when they were, in fact, generated by user B.

### 2.2 Requirement specification

Based on the threat analysis and usecase study, we have specified a set of requirements for an effective, secure and efficient tagging method. The requirements can be classified into three groups, functional, security and performance requirements.

(F) Functional Requirements: Two functional requirements are specified, F1 and F2.
(F1) Public and Private Verifiability: The method should support both public and private verifiability, i.e. to allow both a TPA and the owner of data to verify the integrity of data.
(F2) Dynamic Tag Support: The method should minimise the number of tags that need to be modified or re-computed when any changes are being made to a data file. When some data blocks in a file are modified, new data blocks are inserted, or obsolete data blocks are deleted, the associated tags should also be changed. Such changes should be kept as small as possible.
(S) Security Requirements: Five security requirements are specified.
(S1) Tag Forgery Resistance: It should be computationally infeasible for a PCS provider to forge a tag for some data, which could produce a positive tag verification result.
(S2) Tag Collision Resistance: It should be computationally infeasible to generate identical tags for different data blocks that are owned by the same PCS user or tags generated for the same data blocks but owned by different users should be different too. This is for countering tag replace attacks that may be launched by a PCS provider.
(S3) Non-repudiation of Tag Generation: It should be computationally infeasible for a PCS user to falsely deny that she/he has generated a valid tag for a data block(s).
(S4) Data Confidentiality Preservation: The method should allow the PCS provider and a TPA to verify the validity of a tag without them accessing plaintext data blocks. This requirement is for preserving the confidentiality of data while providing the DIA service.
(S5) Unbounded Verifiability: The method should be such that the security level of a tag is independent of the number of times the tag has been verified. In other words, the verification of tags should not make them more vulnerable to security attacks.
(E) Performance Requirements: Two performance requirements are specified.
(E1) Minimising Tag Generation Cost: The cost incurred in tag generation should be as low as possible.
(E2) Minimising Tag Verification Cost: The cost incurred in tag verification should be as low as possible.

## 3 Related work

This section provides an overview of related tagging methods published in the literature. Depending on the tagging methods, one or more tags may be generated for a single data file. If one tag is generated for a data file, then when verifying the tag (i.e. the integrity of the file), the entire file has to be downloaded from the PCS server. This could be costly in terms of bandwidth, particularly if the file size is large. To reduce this cost, a fragmentation approach is used. With this approach, each data file is divided into multiple data blocks, and a tag is either generated by using multiple data blocks, i.e. the so-called *One Tag for Multiple data Blocks* (OTfMB) approach, or by generated by using a single data block, the *One Tag for a Single data Block* (OTfSB) approach. Depending on which of these two approaches they use, existing tagging methods can largely be classified into two groups: OTfMB based methods and OTfSB based methods. Table 1 provides a summary of the advantages and disadvantages of existing works.

**Table 1:** Advantages and disadvantages of existing methods.

Tagging Methods	Advantages	Disadvantages
Ateniese_1 [3]	Less computational cost in a tag verification	High storage cost Support bounded verifiability (venerable to replay attacks) Support private verification only Venerable to repudiation of tag generation attack
Chen [4]	Less storage and communication costs	Support bounded verifiability (venerable to replay attacks) Support private verification only Venerable to a repudiation of tag generation attack
Krishra [5]	Less computational cost in tag generation and verification attacks	Support bounded verifiability (venerable to replay attacks) Support private verification only Venerable to repudiation of tag generation attacks
Luo_1 [6]	Support batch verification Support unbounded verification	Venerable to replace attacks Support private verification only Venerable to repudiation of tag generation attacks
Sookhak [7]	Support batch verification Support unbounded verification Resist to replace attacks	Support private verification only Venerable to repudiation of tag generation
Ateniese_2 [8]	Support public verification Support non-repudiation of tag generation Support data confidentiality against provider	High computational cost in tag generation and in private tag verification High storage and communication cost Not support dynamic data High storage and communication
Ni [9]	Support public verification Support non-repudiation of tag generation	High storage and communication costs Support static data
Erway [23]	Support data confidentiality against provider Support public verification Support non-repudiation of tag generation	High storage and communication costs Support static data
Hanser [10]	Less storage and communication costs Support public verification Support non-repudiation of tag generation	High computational cost in a private tag verification
Li [11]	Less storage and communication costs Support public verification Support non-repudiation of tag generation	Support static data High computational cost in a private tag verification
Liu [12]	Support public verification Support Non-repudiation of tag generation	High computational cost in a private tag verification
Wang [17, 30, 31]	Support public verification Support non-repudiation of tag generation	Support static data High computational cost in a private tag verification
Yang [32]	Support public verification Support non-repudiation of tag generation	Support static data High computational cost in a private tag verification
Luo_2 [13]	Support public verification Support non-repudiation of tag generation	High computational cost in a private tag verification Support static data
Salim [13]	Support public verification Support non-repudiation of tag generation	High computational cost in a private tag verification Support static data

### 3.1 OTfMB based methods

A file to be integrity protected is usually divided into multiple blocks. With the OTfMB approach, each tag is generated using two or more data blocks, and these data blocks are randomly selected from the blocks of the file. The number of tags that are generated for a file is dependent on the number of data blocks the file has and the number of data blocks that are used in generating each tag. The more the data blocks a file has and/or the fewer the blocks that are used in generating each tag, the more the tags that will be generated for the file.

Each integrity verification of a file typically involves the random selection and verification of one tag of the file. Only the selected tag along with the data blocks that are used to generate the tag will need to be downloaded from the PCS server when carrying out the verification. As the downloaded data blocks is a subset of the data blocks a file has, this approach is cheaper, in terms of bandwidth cost, than the non-fragmentation approach.

The two most notable OTfMB-based tagging methods are those proposed in [3, 4]. The two methods differ in the cryptographic algorithms used. In the method proposed by Ateniese et al. [3] (hereafter referred to as the Ateniese_1 method), a conventional hash function, such as MD5 and SHA, and a symmetric cipher, such as AES, are used. To generate a tag for a file, a randomly selected subset of data blocks are concatenated and hashed. The hash value is then encrypted using a symmetric key. To verify the integrity of a file, a tag is randomly selected from the tags of the file, and a fresh hash value is generated based on the data blocks associated to the tag. The fresh hash value is then compared with the one decrypted from the selected tag. If the two hash values are equal, then the integrity of the file is said to be assured. In this method, the symmetric encryption is used to protect the tags against forgery attacks. As the symmetric key should only be known to the PCS user, it is computationally hard for any unauthorised entities to make any alteration to, or forge, the data file or the tag, such that a freshly computed hash value is identical to the one recovered from the downloaded tag.

The Ateniese_1 method does not support dynamic tagging efficiently. When there is a change in a data file, the associated tag needs to be recomputed from scratch. To improve on this, Ateniese et al. revised their method by replacing the concatenation operation with an XOR operation. In this way, when new data blocks are added, only the XORing operations involving the hash values of the new data blocks and the encryption operation would need to be re-computed.

Longer tags impose a higher storage requirement and also consume more bandwidth when they are downloaded from the server. To reduce the overhead costs in DIA, Chen et al. [4] (the Chen method) proposed to use the algebraic signature function [15] to replace the conventional hash function in the Ateniese_1 method. The algebraic signature function, which is, sometimes, also referred to as an algebraic hash function, differs from a conventional hash function in the following three aspects. Firstly, it is faster to compute. According to [16], it takes about half of the time SHA-1 takes to generate a tag. Secondly, it generates a shorter hash value (thus a shorter signature or tag) than SHA-1. For example, a hash value produced by an algebraic signature function [15] is 16 bits long, which is one-tenth of the size (160 bits) generated by SHA-1. Thirdly, it has an algebraic property that allows multiple signatures to be aggregated in a numerical manner, rather than a simple concatenation of multiple hash values as in the case of conventional hash functions. This signature aggregation property can be exploited to support batch verification of multiple tags so that the verifications of multiple signatures can be carried out by verifying a single aggregated signature.

The above two methods also differ in the size of the verification data, i.e. the data that is used for verifying a tag, which is also the data that is transmitted from the PCS server to a PCS user upon the receipt of a file integrity verification request. With the Ateniese_1 method, the size of the verification data is dependent on the size of a hash value which, in turn, is dependent on the hash function used, or the data blocks number used in a tag generation in case of the PCS user who is generating the hash value, whereas, with the Chen method, it is dependent on the size of a data block; it increases linearly with the data block size.

A major limitation with the OTfMB approach is that, if there are too few tags for a file, e.g. if a file is short and/or if too many blocks are used for each tag generation, the approach is vulnerable to replay attacks. This is because, repeated integrity verifications of the file will lead to repeated use of the same tag(s) and the associated data blocks. This will make it easier for the PCS provider to guess or cache the hash values, or the sum of the hash values, of the data blocks and their associated tags. When a verification request is received, the PCS provider could just dispatch the cached values and the tags, rather than what are actually stored in the file storage. In such cases, unauthorised alterations made to a data file and its tags may go undetected.

To make the guesses harder, or to give a stronger resistance to replay attacks, more tags should be generated for each file. In an extreme case, one tag is generated for each data block in a file, i.e. only one data block is used in each tag generation. This leads to the OTfSB approach. With this approach, for countering replay attacks, each integrity verification can require the use of multiple tags and these tags are typically randomly selected from the whole set of the tags for the file. Obviously, the more tags that are generated for each file, the harder it is for the PCS provider to guess the subset of tags that may be selected for an integrity verification; thus the harder it is to launch a successful replay attack, and the stronger the unbounded verifiability of the method. For this reason, most of the existing methods use the OTfSB approach.

### 3.2 OTfSB based methods

Krishra et al. [5] proposed tagging method (Krishra Method), which is one of OTfSB based methods. The method uses symmetric encryption algorithm to compute tags. For each tag, random bits of its associated data block are encrypted. In each verification, the positions of the random bits are disclosed to the provider to retrieve their values and their associated tag. However, the method can save the cost at the user and provider, but it still cannot support unbounded verifiability even it is based on the OTfSB approach. In each time, the positions of the random bits can be disclosed to the provider. Therefore, tagging methods described in [6–12, 17–19] have been proposed, where a whole block content is used in a tag generation. Depending on the cryptographic algorithms used, these methods can be further classified into algebraic signature based, MAC based, and digital signature based methods.

As mentioned above, an algebraic signature function [15] takes shorter time to generate a signature (tag), and it also generates shorter signatures, in comparison with a conventional hash function. In addition, an algebraic signature function has an additive homomorphic property, i.e. a signature of the sum of multiple data blocks is equivalent to the sum of the signatures of the corresponding data blocks. By using an algebraic signature function, we can generate homomorphic verifiable tags, so for integrity verification, only the sum of the requested data blocks and the sum of the tags corresponding to the data blocks need to be downloaded. The computational and communication costs in DIA are, therefore, independent of the number of data blocks used in verifying a tag. For this reason, it is a popular method used for tagging method designs. The tagging methods proposed by Luo et al. [6] (the Luo_1 method) and by Sookhak et al. [7] (the Sookhak method) are based on an algebraic signature function [15].

Once tags are generated using the Luo_1 method [6], the PCS user in DIA uploads the data blocks onto the PCS server but should keep their associated tags in the local storage for enhancing the security level. When verifying the integrity of the file, the sum of the random data blocks, i.e. a data value, (each block is encoded into a numerical value) and their associated tags are taken as inputs. Then, the algebraic signature function is applied to the data value to generate a fresh algebraic signature (i.e. a fresh tag) and compares it with the one that is computed using the corresponding tags stored in the local storage to see if the two values are equal. However, no measure has been taken to address the issue of tag collisions. If the PCS user in DIA, for reasons such as short of local storage space, wishes to upload the tags onto the PCS server, the method can be vulnerable to tag collisions, i.e. tags generated for different files owned by the same PCS user or by different PCS users may be identical. Because of this, the DIA is vulnerable to integrity fraud.

To overcome this limitation, Sookhak et al. [7] proposed a revised method (the Sookhak method), in which, a file ID and a block index are used to randomise the input of the tag generation algorithm. In addition, the method uses a sector/block fragmentation idea to optimise the trade-off between security and costs. For a given file size, if a larger data block size is used, fewer data blocks thus fewer tags the file will have. This will reduce the security level and the computational cost but increase bandwidth cost. The idea is to use a larger data block size, but further divide each data block into multiple sectors. For each sector, a tag is generated. The tag for a data block is generated by taking the sum (or the product) of the sector tags in the block. When verifying the integrity of the file, the sum of the sectors is used instead of the sum of the data blocks. As the size of a sector is shorter than the size of a data block, the bandwidth cost is lower in DIA.

Tags that are generated by using such an unkeyed function, i.e., conventional hash functions, are not tamper-proof, so they are only suited to trustworthy environments where integrity drifts are caused by accidental errors or non-malicious intent, such as channel or system errors or innocent human errors. However, in our problem context where data are managed by third parties, data integrity drifts may also be caused by malicious intents. In such cases, tags must be tamper-proof, and this can be done by using a secret key to protect the values produced by an unkeyed function. The secret key can either be a symmetric key (of a symmetric-key cipher) or a private key (of a public-key cipher).

Symmetric key based tagging methods, i.e. [18, 19], are suited to cases where file integrity verifications are performed by PCS users (i.e. data owners) themselves, or PCS users trust their TPAs unconditionally. This is because the same key is used for tag generation and verification. In cases where these two conditions are not satisfied, asymmetric (public and private) keys should be used, leading to the so-called asymmetric key (or public-key) based tagging methods. With such a method, a tag is a digital token signed with a PCS user’s (data owner’s) private key, and the corresponding public key is used to verify the tag. So these tagging methods are also called digital signature based tagging methods. There are a number of digital signature algorithms. The most notable ones are the Rivest–Shamir–Adleman (RSA) algorithm [20], Elliptic Curve Digital signature algorithm (ECDSA) [21] and Boneh-Lynn-Shacham based (BLS) algorithm [22]. Depending on the digital signature algorithm used, signature based tagging methods can also be classified into three variants, RSA-based, ECDSA-based and BLS-based.

The tagging methods proposed by Ateniese et al. [8] (the Ateniese_2 method), Ni et al. [9] (the Ni method), and Erway et al. [23] (the Erway method) are RSA-based. The Ateniese_2 method and the Erway method encrypt the data file before fragmenting into data blocks for data confidentiality preservation. Furthermore, they use random number and a data block index in each tag generation for tag collision resistance, whereas the Ni method uses a file ID in addition to a random number and a data block index for collision resistance. Also, the Ni method and the Erway method use the blocks/sector fragmentation idea as described in the Sookhak method above to optimise the trade-off between cost and security.

The RSA algorithm consists of modular exponentiation and inversion operations, so it is relatively expensive in terms of computational complexity and time it takes to generate and verify a tag. The computational cost increases sharply as the size of the key increases [24–27]. The average time it takes for the RSA-1024 algorithm (RSA algorithm with 1024-bit key size) to generate a tag is about 81 milliseconds, and this time increases to about 1254 milliseconds with RSA-2048 [28]. According to the NIST recommendations [29], RSA-2048 should be used for an enhanced level of security. In addition to the high computational cost, the RSA algorithm is also relatively more expensive in terms of storage and communication bandwidth cost; the tag size is 1024 bits with RSA-1024, and 2048 bits with RSA-2048.

With the same security level, the ECDSA algorithm [21] costs less to generate tags and generates shorter tags than the RSA algorithm [24, 25]. For example, it takes about 41 milliseconds for the 192-ECDSA algorithm to generate a tag of 192-bits, and 45 milliseconds for 224-ECDSA to generate a tag of 224-bits. For these reasons, Hanser et al. [10] proposed to use the ECDSA algorithm for tagging method design.

To further reduce the overhead costs, BLS-based tagging methods were proposed and the most notable ones are by Li et al. [11] (the Li method), Liu et al. [12] (the Liu method), Wang et al. [17, 30, 31] (the Wang method), Yang et al. [32] (the Yang method), Luo et al. [13] (Luo_2 method) and Salim et al. [14] (Salim Method). The BLS short signature [22] scheme, as indicated by its name, produces short signatures each having a typical length of 160 bits. This length is shorter than the 192-bits produced by an ECDSA based tagging method and 1024-bits by an RSA-1024 based method. In terms of tag generation cost, according to [24, 25], a BLS-based tagging method has a similar level of cost as an ECDSA-based method. While they are all BLS-based, the methods differ in terms of how the tag collisions are addressed and whether a block/sector fragmentation approach is used. The Li and Wang methods use a data block index to resist tag collisions, whereas the Luo_2 and Salim methods use the hash value of the underlying data block, the Liu method uses, addition to the hash value of the data block, a random number to build collision resistance and the Yang method uses the hash value of a secret hash key, file ID and block index addition to a random number. The Wang and the Luo_2 methods do not use the block/sector fragmentation approach, whereas the Li, Liu, Yang and Salim methods do.

From the above discussions of existing tagging methods, we can make the following observations:

1. None of the existing methods support both public and private verifiability on the same platform in an efficient manner. Symmetric key based methods can only be used to support private verifiability, making them unsuited to TPA-based DIA or in environments where third parties should not be trusted unconditionally or their actions or services should be held accountable. Although asymmetric key based methods can support both public and private verifiability, they are costly to PCS users, particularly if he has a high number of files in PCS.
2. There is still room for improvements with regards to protecting data confidentiality in the design of tagging methods. Some of the existing DIAs were designed under the assumption that TPAs are trustworthy, so the data confidentiality requirement was not considered when the methods were designed. To satisfy this requirement, a few DIAs use a random masking technique. The random masking method disguises the content of data blocks when they are being released from the PCS upon the receipt of an integrity verification request. The masking operation needs to be carried out by the PCS provider whenever a file integrity verification request is received. This imposes an additional run-time overhead to the PCS provider. Also, this approach does not protect data confidentiality against PCS providers. On the other hand, some the tagging methods that are designed to support data confidentiality requirement against PCS provider and TPA, they use encryption at a file-level, where a data file is encrypted and then divided into multiple blocks. Unfortunately, by using these methods, the DIAs cannot support the dynamic tag efficiently, where a high computational cost can be introduced at the PCS user.
3. None of the existing methods can support dynamic tag efficiently and provide tag collision resistance at the same time. Some of the methods using a data block index and/or file ID for the collision, but this leads to incur a computational cost at the PCS user in updating tags. On the other hand, other methods used a hash value of the data block for tag collision resistance and dynamic tags. Unfortunately, they are not considering the collision between multiple PCS users.

The novel tagging method reported in this paper is designed to overcome these limitations, and, in addition, the method is designed to be cost-efficient, i.e. imposing as less overhead costs as possible. In the remaining part of this paper, we present the design, analysis and evaluation of this method, i.e. the TOD method.

## 4 The TOD method

This section describes our novel TOD method. It first gives the design preliminaries, covering assumptions and notations. It next presents the key features and the building blocks that are used in the design, before describing the TOD method in detail.

### 4.1 Design preliminaries

#### 4.1.1 Assumptions

As the focus of this work is on the design of a tagging method, the following assumptions are used in the security analysis of the method.

(A1) All the cryptographic algorithms used, including the pseudo-random number generator, are secure.
(A2) Cryptographic keys are securely generated, distributed and stored.
(A3) The focus of this work is on tackling insider threats in relation to data integrity. Some of the external attacks, such as impersonation, are outside of the scope of this work. In other words, communication channels linking the DIA-ETTP entities are assumed to be authenticated. This can be achieved by using off-the-shelf technologies such as a Secure Socket Layer (SSL).

#### 4.1.2 Notations

The notations used in the remaining part of this paper is summarised in Table 2.

**Table 2:** Notations used in the design of TOD.

Symbol	Meaning
*DF*	Data file.
*DB*_*i*_	*i*^*th*^ data block in a data file.
{*DB*_*i*_}	Set of the data blocks.
*K*	Total number of data blocks in a data file.
*S*	Total number of sectors in a data block.
*T*	Number of data blocks used in one tag generation (In the OTfSB approach, T is equal 1).
*NT*	Number of required tags are generated for one data file (In the OTfSB approach, *NT* is equal to *K*, the total number of data blocks in a file).
*C*	Number of data blocks used in each verification.
*d*	Total number of data blocks in a file after eliminating redundant data blocks, where 1 ≼ *d* ≼ *K*.
{0, 1}*	Set of bit strings.
{0, 1}^*n*^	Set of bit strings of length n.
*L*_*a*_	Bit-length of *a* where *a* ∈ {0, 1}*.
*Z*_*p*_	Set of positive integers modulo a large prime p.
*a* ←_*R*_ *A*	Randomly and uniformly chosen element *a* from a finite set *A*.
*sk*	User’s LiSHE secret key
*ppk*_*En*_	User’s Paillier public key
*x*	User’s BLS private key
*ppk*	User’s BLS public key
*User*_*ID*_	ID of the owner of the file.
*RN*_*i*_	Random number generated using a secure pseudorandom number generator.

### 4.2 Key features and ideas

The TOD method has five features, and three of these features are novel. This section gives these features along with the ideas used to achieve the features.

1. TOD supports both public and private verifiability on the same platform efficiently. For to make DIA more secure, it should not assume that any of the third parties involved in managing and/or verifying users’ data is trustworthy. TOD achieves this by supporting both public and private verifiability so that routine, or more frequent, verifications of data integrity can be delegated to a third party, TPA, but the owner of the data can also verify the integrity of their data anytime they wish. In this way, we can shift the burden of data integrity verifications away from data owners, while, at the same time, giving data owners the option of monitoring the services provided by the third parties by equipping them with the ability to detect any integrity drift that may be caused by the PCS provider and/or any forgery of integrity verification results by the TPA. In other words, by supporting the dual verifiability, we make the integrity protection more effective, protecting against threats from not only external entities but also authorised insiders. This feature is achieved through a hybrid use of two cryptographic functions. The former supports public verifiability and non-repudiation of tag generations, while the latter supports private verifiability.
2. TOD supports integrity verification of both plaintext data and ciphertext (encrypted) data. Tag verifications can be carried without the need to decrypt any encrypted data. This feature can help to preserve data confidentiality while supporting data integrity and dynamic data in a more efficient manner. This is part of the measure to reduce trust on the third parties. This feature is provided by using a homomorphic encryption scheme to encrypt any data block, that used in tags generation, uploaded onto PCS.
3. TOD supports tag deduplication. Tag deduplication means that a single tag can be used to authenticate multiple copies of the same data can be authenticated by using a single tag. This can reduce the number of tags generated, thus reducing computational and storage overheads.
4. TOD achieves tag collision resistance without coupling the tags and files are used to protect. This decoupling allows tag deduplication and also allows us to support dynamic data more efficiently. To achieve collision resistance, we use a PCS user ID along with a random number, alternative of using data block index or a file ID. In this way, different tags for the same file or different files are completely decoupled. If one tag is to be updated, other tags will not be affected. Furthermore, for identical data blocks that appear in multiple files, only one tag needs to be generated. This can help to reduce the number of tags generated across all the files a user has on the PCS, further reducing computational and storage overheads.
5. TOD is designed to achieve the above properties with as less overhead costs (computational, storage and communication costs) as possible, especially for the user end. This is done by taking the following two measures. The first is, we have chosen to use more efficient signature functions, the algebraic signature and BLS signature function, to achieve the property of private and public verifiability. The algebraic signature and BLS signature functions generate shorter tags, and are also computationally cheaper than other signature functions. The second measure is to use signature aggregation in supporting private and public verifiability, allowing a PCS user and TPA to verify multiple tags in one operation, thus reducing verification costs imposed on the PCS user and TPA.

### 4.3 Cryptographic building blocks

The design of the TOD method has made use of four cryptographic schemes as its underlying building blocks. The schemes are the LiSHE (it is a symmetric key based additive homomorphic encryption scheme) [33], the Paillier (it is an asymmetric key based additive homomorphic encryption scheme) [34], the algebraic signature [15] and the BLS [22]. The LiSHE scheme is used for protecting the confidentiality of data files, while the other three schemes are for the generation and verification of tags. In the following, we give an overview of these schemes and justifications for their selections.

Homomorphic encryption is a type of encryption algorithm [35–37] that allows computation to be carried out on ciphertext data, thus preserving the confidentiality of data while them being computed. There are two types of homomorphism, additive homomorphism and multiplicative homomorphism. In this work, we need additive homomorphism. An encryption scheme is said to be additively homomorphic if the encryption of the sum of two (or more) plaintext data blocks is equivalent to the sum of the ciphertexts of the corresponding data blocks. Mathematically, this can be expressed as: *HE*(*DB*_1_ + *DB*_2_) = *HE*(*DB*_1_) + *HE*(*DB*_2_), where *DB*_1_ and *DB*_2_ are two plaintext data blocks, HE denotes the additive homomorphic encryption scheme, and ‘+’ addition operation.

Depending on the types of keys used, a homomorphic encryption scheme can be either a Symmetric Homomorphic Encryption (SHE) scheme or an Asymmetric Homomorphic Encryption (AHE) scheme. A SHE scheme uses the same key for encryption and decryption, whereas an AHE scheme uses two different keys, one for encryption and the other for decryption. To the best of the authors’ knowledge, there are four SHE schemes published in the literature, and these are respectively proposed by Li et al. [33], Dasgupta et al. [38], Chan et al. [37] and Xiao et al. [39]. With regard to AHE schemes, the most popular ones are the RSA [20] and Paillier [34] scheme. The RSA scheme supports multiplicative homomorphism, whereas the Paillier scheme supports additive homomorphism.

Generally, SHE schemes are computationally cheaper than AHE schemes. As shown in Table 3, for encryption, the SHE scheme, proposed by Li et al. (hereafter referred to as the LiSHE scheme) uses one exponentiation operator, whereas the Paillier scheme uses two exponentiation operators. But an SHE scheme does have downside, i.e. the need for the key distributions. However, this is not an issue for data files encryptions in our problem context, as a data file is both encrypted and decrypted by the same entity, i.e. its data owner (PCS user). For these reasons, we have decided to use an SHE scheme for confidentiality protection of PCS users’ data files.

**Table 3:** Computational complexities of the Paillier, RSA and LiSHA schemes.

	Paillier	RSA	LiSHA
Encryption Complexity	2 $Exp_{Z_{n^{2}}}$	$Exp_{Z_{n}}$	$Exp_{Z_{p}}$
Decryption Complexity	$Exp_{Z_{n^{2}}}$	$Exp_{Z_{n}}$	$Exp_{Z_{p}}$

*Exp*_*x*_: Modular exponentiation in *x*

The next question is which SHE scheme we should go for. Among the four known SHE schemes [33, 37–39], the LiSHE scheme, proposed by Li et al. [33], is the most efficient one. The scheme is based on integer operations (with the computational complexity of matrix operations), which is computationally cheaper than the matrix multiplication and matrix inversion operations used in the schemes designed by Chan et al. [37] and Xiao et al. [39] (the computational complexity of a matrix multiplication operation is O(*n*^3^) for multiplying two matrices of size (*n* × *n*) [40]). With regard to the SHE scheme proposed by Dasgupta et al. [38], a bootstrapping process is required after a certain number of addition/ multiplication operations to ensure that ciphertexts can be decrypted correctly. This requirement is not desirable and also the bootstrapping process imposes additional overhead.

The Paillier scheme [34] is chosen because it is an asymmetric key based and supports additive homomorphism. The algebraic signature scheme [15] allows signature aggregation and aggregated signature verification. The BLS scheme [22] is the most efficient signature scheme. So these schemes are selected to support public and private verifiability in a secure and efficient manner.

#### LiSHE scheme

The LiSHE scheme consists of three algorithms, a key generation algorithm (LiSHE-KeyG) for generating a symmetric key used to encrypt and decrypt data files, an encryption algorithm (LiSHE-Enc) for encrypting plaintext data files, and a decryption algorithm (LiSHE-Dec) for decrypting ciphertext data files. The details of these algorithms are given below.

**LiSHE-KeyG algorithm**: Given a security parameter, λ, this algorithm generates a secret key, *sk* = (*s*, *q*), and a public parameter, *p*, where *q* and *p* are prime numbers, *p* ≫ *q*, ′≫′ denoting *p* should be much greater than *q*, i.e. the length of *q*, *L*_*q*_ ≥ λ bits, and length of *p*, *L*_*p*_ = 120 × *d* + *L*_*q*_ bits, *d* is a small positive integer called ciphertext degree and *s* is a random number from $Z_{p}^{*}$.

**LiSHE-Enc algorithm**: Given *sk* and a plaintext data block (*DB*) ∈*F*_*q*_, choose a number, *r*, where *r* is a large random positive integer called random ingredient of ciphertext, encrypt the data block to produce the ciphertext output, *c*, as:

$$\begin{array}{cc} c & =\text{LiSHE-Enc}(sk,DB)\\ & =s^{d}\times \left(r\times q+DB\right)\;\text{mod}\;p \end{array}$$ (1)

**LiSHE-Dec algorithm**: Given *sk*, a ciphertext, *c*, and *d*, recover the plaintext data block, *DB*, from the ciphertext, *c*, as:

$$\begin{array}{cc} DB & =\text{LiSHE-Dec}(sk,c,d)\\ & =(c\times s^{-d}\;\text{mod}\;p)\;\text{mod}\;q \end{array}$$ (2)

#### Paillier scheme

The second homomorphic encryption scheme used in the TOD design is the Paillier scheme which is an asymmetric additive HE scheme. The Paillier scheme consists of three algorithms, a key generation algorithm (Paillier-KeyG) for generating a pair of keys, a public key for encryption and a private key for decryption, an encryption algorithm (Paillier-Enc) for encrypting plaintext data, and a decryption algorithm (Paillier- Dec) for decrypting ciphertext data. The details of these algorithms are given below.

**Paillier-KeyG algorithm**: Given two prime numbers, *p* and *q*, this algorithm generates a public key, *ppk*_*En*_ = (*n*, *g*), and a private key, *pk*_*D*_ = (λ, *μ*), where *n* = *p* × *q*, and *g* is an random integer, and $g\in Z_{n^{2}}^{*}$. λ = *lcm*(*p* − 1, *q* − 1), where *lcm* means least common multiple, and *μ* = (*L*(*g*^λ^ mod *n*^2^))^−1^ mod *n*, where $L\left(x\right)=\frac{x-1}{n}$.

**Paillier-Enc algorithm**: Given a public key, *ppk*_*En*_, and a data block (i.e. the message to be encrypted), *DB*, where 0 ≤ *DB* < *n*, select random integer, *r*, where 0 < *r* < *n* and $r\in Z_{n^{2}}^{*}$, encrypt the message, *DB*, to produce the ciphertext output, *c*, as:

$$\begin{array}{c} c=E\left(DB,ppk_{En}\right)=g^{DB}\times r^{n}\;\text{mod}\;n^{2} \end{array}$$ (3)

**Paillier-Dec algorithm**: Given a private key, *pk*_*D*_, a ciphertext, *c*, recover the plaintext message, *DB*, from the ciphertext, *c*, as:

$$\begin{array}{c} DB=D\left(c,pk_{D}\right)=L\left(c^{\lambda }{\;\text{mod}\;n}^{2}\right)\times \mu \;\text{mod}\;n \end{array}$$ (4)

As mentioned earlier, the Paillier scheme supports the additive homomorphism. This means that, given ciphertexts of *DB*_1_ and *DB*_2_, one can compute the ciphertext of *DB*_1_ + *DB*_2_, i.e. the following equation holds:

$$\begin{array}{c} E\left(DB_{1}+DB_{2}\right)=E\left(DB_{1}\right)\times E\left(DB_{2}\right) \end{array}$$ (5)

#### Algebraic signature scheme

The third cryptographic building block used in the TOD design is the algebraic signature function proposed by Thomas Schauer et al. [15]. This function is defined in a Galois field (*GF*(2^*m*^)). For a data block (*DB*) consisted of *w* *m*-bit binary strings, {*s*_*i*_}, 0 ≼ *i* ≼ *w* − 1, its algebraic signature is calculated as:

$$\begin{array}{c} AS\left(DB\right)=\sum_{i=0}^{w-1}s_{i}\times \alpha^{i} \end{array}$$ (6)

where *α* is a primitive element of *GF*(2^*m*^).

The length of a signature generated by this function is equal to the length of *α*, which is an element in *GF*(2^*m*^). For example, using *GF*(2^16^), where the length of *α* is 16-bits, the resulting signature would be an element in *GF*(2^16^) with a signature length of 16-bits (2 bytes). The algebraic signature scheme is a type of hash function with an algebraic property: a signature of the sum of data blocks is equivalent to the sum of the signatures of the corresponding data blocks, i.e., *AS*(*DB*_1_) + *AS*(*DB*_2_) = *AS*(*DB*_1_ + *DB*_2_).

$$\begin{array}{cccc} AS\left(DB_{1}\right)+AS\left(DB_{2}\right) & =\sum_{i=0}^{w-1}s_{1,i}\times \alpha^{i}+\sum_{i=0}^{w-1}s_{2,i}\times \alpha^{i}\\ & =\sum_{i=0}^{w-1}\alpha^{i}\times \left(s_{1,i}+s_{2,i}\right)\\ & =AS(DB_{1}+DB_{2}) & & \end{array}$$ (7)

#### BLS scheme

The fourth cryptographic building block used is the BLS signature scheme [22]. The BLS signature scheme is based on a bilinear pairing, and generates short signatures. In addition, it has an important property, i.e. it allows the aggregation of multiple signatures and the verification of the aggregated signature. In other words, it allows multiple signatures being verified in one operation, the so called batch verifiability property.

The bilinear pairing can be defined as follows. Let *G*_1_, *G*_2_ and *G*_*T*_ be three multiplication cycle groups of prime order *p*, *g*_1_ is a generator of *G*_1_ and *g*_2_ is a generator of *G*_2_. The bilinear pairing is a map *e*: *G*_1_ × *G*_2_ → *G*_*T*_. It has the following properties:

(P1) Bilinear: *e*(*W*^*a*^, *R*) = *e*(*W*, *R*^*a*^) for *W* ∈ *G*_1_, *R* ∈ *G*_2_ and *a* ∈ *Z*_*p*_.
(P2) Non-degeneracy: *e*(*g*_1_, *g*_2_) ≠ 1.

Given the bilinear pairing definition, the BLS signature scheme can be defined as follows. Let (*G*_1_, *G*_2_, *G*_*T*_, *g*_2_, *p*, *e*, *H*()) be the system parameters, where *G*_1_, *G*_2_, *G*_*T*_, *g*_2_, *p*, *e* have been defined above, and *H*() is a BLS hash function, *H*() = {0, 1}* → *G*_1_. The BLS signature scheme consists of three algorithms: a key generation algorithm (BLS-KeyG) for generating signature signing and verification keys, a signature generation algorithm (BLS-SigG) for generating a BLS signature, and a signature verification algorithm (BLS-SigV) for verifying the signature.

**BLS-KeyG algorithm**: Select a random number, *x*←_*R*_ *Z*_*p*_, where *x* is the private key, and compute the corresponding public key (*ppk*), where $ppk=g_{2}^{x}$.

**BLS-SigG algorithm**: Given a data block, *DB* ∈ {0, 1}*, and a private key, *x*, compute a signature, *DBSig*, for the data block, *DB*, where *DBSig* = BLS-SigG (DB) = *H*(*DB*)^*x*^ and *DBSig* ∈*G*_1_.

**BLS-SigV algorithm**: Given a data block, *DB*, its signature, *DBSig*, and the public key, *ppk*, compute and verify if this equation holds, i.e., *e*(*DBSig*, *g*_2_) = *e*(*H*(*DB*), *ppk*).

The BLS signature scheme can be extended into an aggregated signature scheme by which multiple BLS signatures can be aggregated into a single aggregated signature, and the verifications of the multiple signatures are transformed into the verification of the aggregated signature. This aggregated signature scheme consists of four algorithms: a key generation algorithm (the BLS-KeyG algorithm) for generating signature signing and verification keys; a BLS signature signing algorithm (the BLS-SigG algorithm) for generating a BLS signature, a signature aggregation algorithm (the BLS-AggSigG algorithm) for aggregating multiple BLS signatures into a single aggregated BLS signature, and an aggregated signature verification algorithm (the BLS-AggSigV algorithm) for verifying the aggregated signature. The BLS-KeyG and BLS-SigG algorithms are defined above, the BLS-AggSigG and BLS-AggSigV algorithms are defined below.

**BLS-AggSigG algorithm**: Given *w* BLS signatures, i.e. {*DBSig*_*i*_}, where, 0 ≼ *i* ≼ *w*−1, each signed on a distinct data block, {*DB*_*i*_}, using the BLS-SigG algorithm, this algorithm generates an aggregated BLS signature, *AggDBSig*, using the equation:

$$\begin{array}{c} AggDBSig=BLS-AggSigG\left(\left\{DBSig_{i}\right\}\right)=\prod_{i=0}^{w-1}DBSig_{i}. \end{array}$$

**BLS-AggSigV algorithm**: Given an aggregated signature, i.e. *AggDBSig*, a public key, *ppk*, and *w* data blocks, {*DB*_*i*_}, that have been signed, where {0, *w* − 1}. This algorithm verifies the aggregated signature by computing a hash value for each of the *w* data blocks, i.e. *H*(*DB*_*i*_), where *i* ∈ {0, *w* − 1}, and confirming if this equation holds, *e*(*AggDBSig*, *g*_2_) = $e(\prod_{i=0}^{w-1}H\left(DB_{i}\right),ppk)$. If yes, the aggregated signature is accepted. Otherwise, it is rejected.

### 4.4 The TOD method in detail

**Fig 2:**
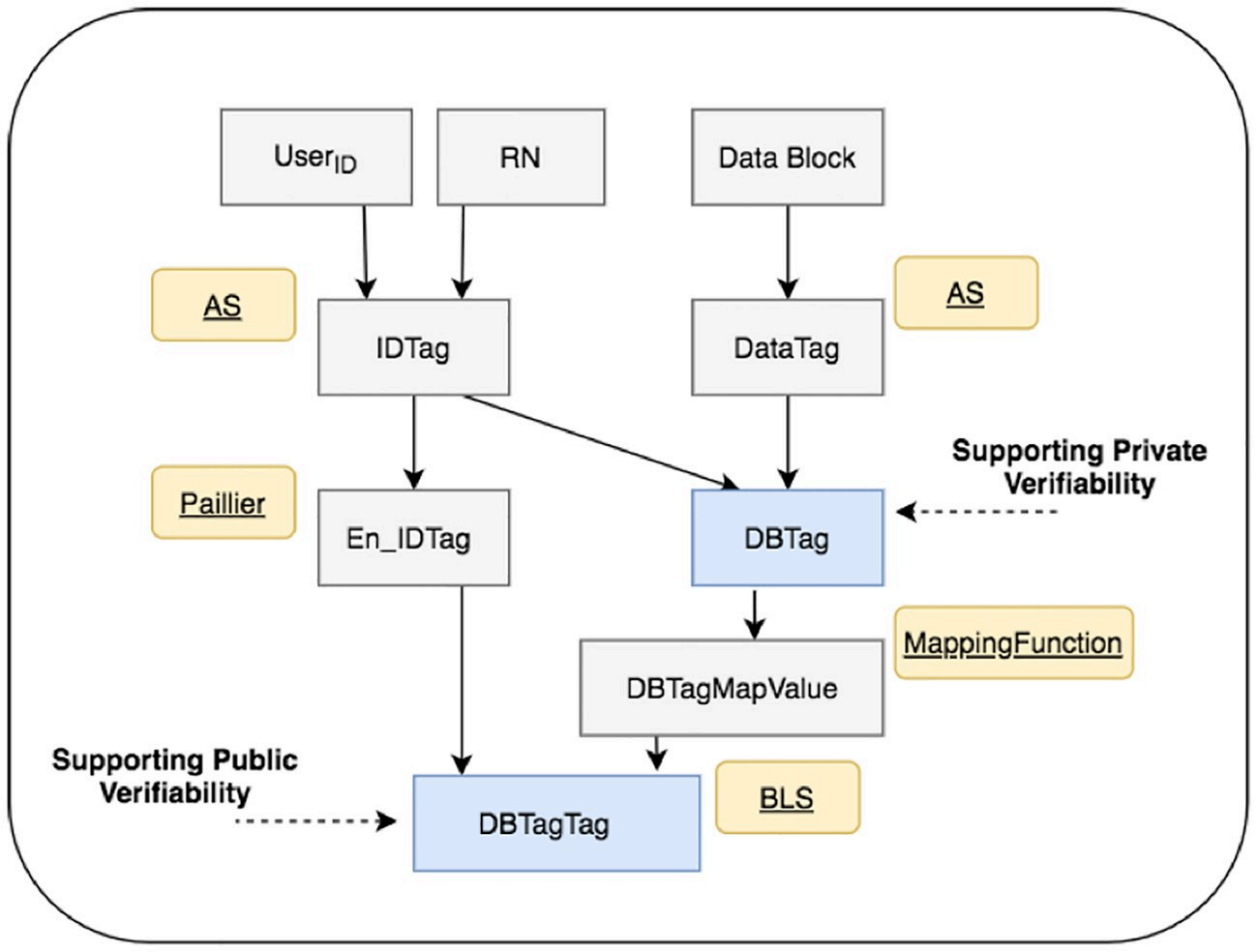
Four types of tags, their relationship and inputs and schemes used to generate them.

A major novelty of the TOD method lies in that it supports both private and public verifiability securely and efficiently. This means that, once tags are generated for a file, both the owner of the file and a third party representing the owner can do the integrity verification of the file securely and independently at any frequencies. This property is achieved by using four types of tags that are generated and secured by making a hybrid use of the algebraic signature (AS) scheme, a MappingFunction, the BLS signature scheme, and the Paillier scheme. The four types of tags are, respectively, an identifier tag (*IDTag*), a data tag (*DataTag*), a data block tag (*DBTag*), and a *DBTag* tag (*DBTagTag*). Fig 2 shows the relationship of these tags, and the input and the scheme that are used for generating each.

The *IDTag* serves as an identifier for differentiating different DBTags. It is also used as an input for the generation of *DBTags* to resist tag collisions. *IDTags* are generated by using the AS scheme which defined in Eq (6), along with two parameter values, the ID of the user (i.e. the owner of the data block) and a random number that is unique for each *IDTag*. In this way, any change made to a data file would only affect the tag(s) of the data block(s) that have been affected by the change. This can reduce tag generation overhead. In addition, in our design, the *IDTags* are encrypted. The encryption is done by using the Paillier scheme to protect the confidentiality of the *IDTags* to counter potential *IDTag* forgeries by authorised insiders, i.e. the PCS provider or the TPA. The DataTag of a data block represents the digest of the data block, whereas the *DBTag* of a data block is the digest of the *IDTag* and *DataTag* associated to the data block. Similar to the case for *IDTags*, *DataTags* and *DBTags* are also generated by using the AS scheme. The *DBTags* of a data file are used to support the private verifiability of the data file.

The *DBTagTag* of a data block provides an extra layer of protection protecting the integrity of *IDTag* and *DBTag* associated to the data block against fraud that may be committed by authorised insiders (the PCS provider and the TPA). It is generated by using the BLS scheme on an encrypted form of the *IDTag* and the *DBTag*. *DBTagTags* of a data file are used to support the public verifiability of the data file.

**Algorithm 1**: SetUp

**Input**: *DF*, *sk*

**Output**: {*En*_*DB*_*i*_}, 0 ≼ i ≼ *d* − 1

  1. Divide a data file (*DF*) into *K* data blocks, {*DB*_*i*_}, 0 ≼ *i* ≼ *K* − 1.

  2. Eliminate any additional identical data blocks among *K* data blocks, i.e. only keep one copy of any identical blocks. The output of this step is *d* non-duplicated data blocks, {*DB*_*i*_}, 0 ≼ i ≼ *d* − 1.

  3. Encrypt each of *d* non-duplicated data blocks, {*DB*_*i*_}, using the LiSHE-Enc algorithm and a key, *sk*, to produce a set of encrypted data blocks, {*En*_*DB*_*i*_}, 0 ≼ i ≼ *d* − 1.

    **for** *i* = 0 → *d* − 1 **do**

      Compute: *En*_*DB*_*i*_ = LiSHE-Enc(*DB*_*i*_, *sk*)

    **end**

**Fig 3:**
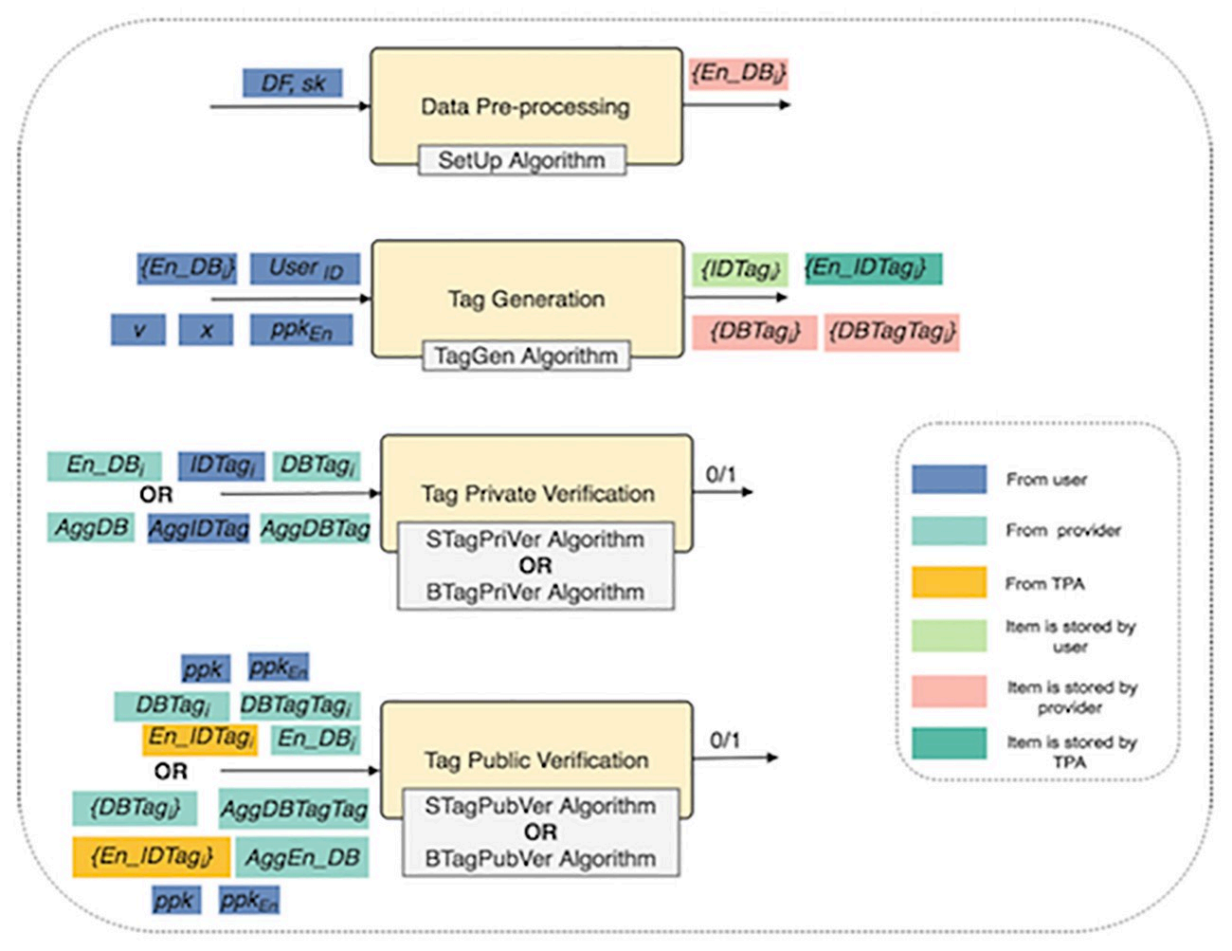
The functional components of the TOD method.

In the following, we describe, in detail, four functional components of the TOD method, namely, data pre-processing, tag generation, tag private verification (for private verifiability) and tag public verification (for public verifiability). Fig 3 shows the functional components of the TOD method and their algorithms (inputs and outputs).

#### Data pre-processing

A data file is first pre-processed before tags are generated for the file. The pre-processing involves fragmenting the data file into multiple data blocks, eliminating any redundant (or duplicated) data blocks producing the so-called non-duplicated data blocks (*DBs*), and encrypting them producing the encrypted data blocks (*En*_*DBs*). Data deduplication is done by comparing data block values and then removing any additional blocks that have identical values. The encryption is done by using the LiSHE-Enc algorithm described in Section 4.3 above. This encryption operation is to protect the confidentiality of the data file ensuring that the content of the data file can only be accessed by the user (i.e. the owner of the file) him/erself even if the file is being managed by third parties. These data pre-processing operations are implemented in the SetUp algorithm. The algorithm takes a data file (*DF*) and a symmetric key, *sk*, as its input and outputs a set of encrypted data blocks {*En*_*DB*_*i*_}.

**Algorithm 2**: MappingFunction

**Input**: *DBTag_i_*, *MappingSecretKey*

**Output**: *DBTagMapValue*_*i*_

  1. Compute: *S* = *DBTag*_*i*_ || *MappingSecretkey*.

  2. Compute:

$$\begin{array}{c} t=H1(S) \end{array}$$ (12)

  3. Convert *t* to integer, *a*.

  4. Compute:

$$\begin{array}{c} DBTagMapValue_{i}=a\;\text{mod}\;p \end{array}$$ (13)

#### Tag generation

As mentioned earlier, each data block has four tags: *IDTag*, *DataTag*, *DBTag*, and *DBTagTag*. The math formulas for the generations of these tags are summarised in Table 4.

**Table 4:** Math equations for the generations of different tags.

Tags	Equations
Identifier Tag	*IDTag_i_* = *AS*(*User_ID_* ‖ *RN_i_*) (8)
Data Tag	*DataTag_i_* = *AS*(*En*_*DB_i_*) (9)
Data Block Tag	*DataTag_i_* = *IDTag_i_* + *DataTag_i_* (10)
DBTag Tag	$\begin{array}{c} DBTagTag_{i}=[H\left(En\_IDTag_{i}\right)\times \upsilon^{DBTagMapValue_{i}}{]}^{x} \end{array}\;(11)$

*En*_*DB*_*i*_ is an encrypted data block, *En*_*IDTag*_*i*_ is an encrypted form of *IDTag*_*i*_, *H*() is a BLS hash function, *υ* is chosen uniformly at random from *G*_1_, *AS*() is the function defined in Eq (6), *DBTagMapValue*_*i*_ is the output from the MappingFunction algorithm, and *x* is the file owner’s BLS private key.

From the Table, it can be seen that an *IDTag*, *IDTag*_*i*_, for a data block, *DB*_*i*_, is generated by applying the concatenation of the user’s ID, *User*_*ID*_, and a random number, *RN*_*i*_ to the *AS* scheme, as shown in EQ (8).

**Algorithm 3**: TagGen

  **Input**: {*En*_*DB*_*i*_}, 0 ≼ i ≼ *d* − 1, *User_ID_*, *v*, *x*, *ppk_En_*

  **Output**: {*IDTag*_*i*_}, {*En*_*IDTag*_*i*_}, {*DBTag*_*i*_}, {*DBTagTag*_*i*_}, 0 ≼ i ≼ *d* − 1

1 **for** *i* = 0 → *d* − 1 **do**

    1. Generate a random number, *RN*_*i*_, using pseudo-random number generator.

    2. Compute *IDTag*_*i*_ by applying *User*_*ID*_ and *RN*_*i*_ to EQ (8).

    3. Compute *DataTag*_*i*_ by applying *En*_*DB*_*i*_ to EQ (9).

    4. Compute *DBTag*_*i*_ by applying *IDTag*_*i*_ and *DataTag*_*i*_ to EQ (10).

    5. Compute *En*_*IDTag*_*i*_ by applying *IDTag*_*i*_ and *ppk*_*En*_ to Eq (3).

    6. Compute *DBTagMapValue*_*i*_ by using *DBTag*_*i*_ and MappingFunction algorithm.

    7. Compute *DBTagTag*_*i*_ by applying *υ*, *x*, *En*_*IDTag*_*i*_ and *DBTagMapValue*_*i*_ to EQ (11).

2. **end**

A *DataTag*, *DataTag*_*i*_, is a signature token on the ciphertext of a data block, *En*_*DB*_*i*_, generated using EQ (9). A *DBTag*, *DBTag*_*i*_, is a tag generated by taking the numeric sum of the *IDTag*, i.e. *IDTag*_*i*_, and the *DataTag*, i.e. *DataTag*_*i*_, of the block, as shown in EQ (10).

The set of data block tags, {*DBTag*_*i*_}, generated for a set of encrypted data blocks, {*En*_*DB*_*i*_}, are for integrity verification of the data blocks by the data owner, i.e. for achieving private verifiability.

*DBTagTags* are for supporting public verifiability. The generation of a *DBTagTag* is by making a hybrid use of the BLS-SigG scheme (defined in Section 4.3 above) and a MappingFunction defined in Algorithm 2. This MappingFunction uses a hash function, *H*1() (e.g. SHA256), to generate a hash value of *DBTag* concatenated with a secret key, *MappingSecretKey*, and then converts the hash value into an element in *Z*_*p*_. In detail, given an encrypted *IDTag*, *En*_*IDTag*_*i*_, a Map value of *DBTag*_*i*_, *DBTagMapValue*_*i*_, a random number from *G*_1_, *υ*, and the file owner’s BLS private key, *x*, the associated *DBTagTag* is generated using EQ (11) as shown in Table 4.

The tag generation methods for all the four types of tags are implemented in the TagGen algorithm (Algorithm 2).

{*IDTag*_*i*_} should be kept secret (known only to the file owner, i.e. the generator of the tags) and their encrypted copies, i.e. {*En*_*IDTag*_*i*_}, can be uploaded onto TPA for public verifiability. The following three sets: {*En*_*DB*_*i*_}, {*DBTag*_*i*_} and {*DBTagTag*_*i*_}, are loaded onto the PCS server. {*En*_*DB*_*i*_}, {*IDTag*_*i*_} and {*DBTag*_*i*_} are used for file integrity private verifications, whereas {*En*_*IDTag*_*i*_}, {*En*_*DB*_*i*_}, {*DBTag*_*i*_} and {*DBTagTag*_*i*_} are used for file integrity public verifications.

#### File integrity private verification

The private verification of the integrity of the data file refers to the verification of the integrity of the data file by the owner of the data file. This is also referred to as tag private verification. The verification can either be performed on per tag basis, in which case, it is called Single Tag Private Verification (STagPriVer), or in an aggregated manner (i.e. multiple tags are verified in one verification operation), in which case, it is called Batch Tag Private Verification (BTagPriVer).

**Algorithm 4**: STagPriVer

**Input**: *En*_*DB_i_*, *IDTag_i_*, *DBTag_i_*

**Output**: 0/1

  1. Compute a fresh *DataTag*_*i*_, $DataTag_{i}^{\prime }$, by applying *En*_*DB*_*i*_ to EQ (9).

  2. Compute a fresh *DBTag*_*i*_, $DBTag_{i}^{\prime }$, by applying *IDTag* and $DataTag_{i}^{\prime }$ to EQ (10).

  3. **if**

$$\begin{array}{c} DBTag_{i}^{\prime }==DBTag_{i} \end{array}$$ (25)

    **then**

      The private verification is positive, i.e. 1.

    **else**

      The private verification is negative, i.e. 0.

    **end**

A tag private verification involves the use of three data items, i.e. *En*_*DB*_*i*_, *IDTag*_*i*_ and *DBTag*_*i*_, in the STagPriVer case, or three sets of items, i.e. {*En*_*DB*_*i*_}, {*IDTag*_*i*_} and {*DBTag*_*i*_}, in the BTagPriVer case. The math formulas for these verifications are summarised in Table 5. In a tag private verification operation, a secret item involved is *IDTag*_*i*_ that should only be known to the verifier, i.e. the user. The rest of the items are fetched from the provider. The verification process of STagPriVer is as follows. The user computes a fresh *DataTag*, $DataTag_{i}^{\prime }$, by applying *En*_*DB*_*i*_ to EQ (9), then computes a fresh $DBTag_{i}^{\prime }$ by applying *IDTag*_*i*_ and $DataTag_{i}^{\prime }$ to EQ (10), and compares the freshly computed $DBTag_{i}^{\prime }$ with the one retrieved, *DBTag*_*i*_. If the two values are equal, then the verification is positive or true (denoted as 1). Otherwise, it is negative or false (0). The algorithm for this verification is summarised in STagPriVer algorithm (Algorithm 4).

**Table 5:** Math equations for tag verifications (private and public).

	Equations
An aggregated *En*_*DB* value of C *En*_*DBs*, {*En*_*DB*_*i*_}	$\begin{array}{c} AggEn\_DB=\sum_{i=0}^{C-1}En\_DB_{i} \end{array}\;(14)$
A tag of *AggEn_DB*	*AggEn_DBTag* = *AS*(*AggEn_DB*) (15)
An aggregated *IDTag* value of C *IDTags*, {*IDTag*_*i*_}	$\begin{array}{c} AggIDTag=\sum_{i=0}^{C-1}IDTag_{i} \end{array}\;(16)$
An aggregated *DBTag* value of C *DBTags*, {*DBTag*_*i*_}	$\begin{array}{c} AggDBTag=\sum_{i=0}^{C-1}DBTag_{i} \end{array}\;(17)$
A fresh value of *AggDBTag*	*AggDBTag*′ = *AggIDTag* + *AggEn_DBTag* (18)
A fresh value of *En_DBTag_i_*	$\begin{array}{c} En\_DBTag_{i}^{\prime }=En\_IDTag_{i}\times En\_DataTag_{i} \end{array}\;(19)$
*DBTagTag_i_* verification	$\begin{array}{c} e\left(DBTagTag_{i},g_{2}\right)=e\left(H\left(En\_IDTag_{i}\right)\times \upsilon^{DBTagMapValue_{i}},ppk\right) \end{array}\;(20)$
An aggregated *En*_*IDTag* value of C *En*_*IDTags*, {*En*_*IDTag*_*i*_}	$\begin{array}{c} AggEn\_IDTag=\prod_{i=0}^{C-1}En\_IDTag_{i} \end{array}\;(21)$
A fresh value of *En_AggDBTag*	*En_AggDBTag*′ = *AggEn_IDTag* × *En_AggEn_DBTag* (22)
An aggregated *DBTagTag* value of C *DBTagTags*, {*DBTagTag*_*i*_}	$\begin{array}{c} AggDBTagTag=\prod_{i=0}^{C-1}DBTagTag_{i} \end{array}\;(23)$
*AggDBTagTag* verification	$\begin{array}{c} e\left(AggDBTagTag,g_{2}\right)=e\left(\prod_{i=0}^{C-1}H\left(En\_IDTag_{i}\right)\times \upsilon^{\sum_{i=0}^{C-1}DBTagMapValue_{i}},ppk\right) \end{array}\;(24)$

**Algorithm 5**: BTagPriVer

**Input**: *AggDB*, *AggIDTag*, *AggDBTag*

**Output**: 0/1

  1. Compute an algebraic signature of *AggEn*_*DB*, producing an aggregated data tag, *AggEn*_*DBTag*, using EQ (15).

  2. Compute a fresh *AggDBTag*, *AggDBTag*′, by applying *AggIDTag* and *AggEn*_*DBTag* to EQ (18).

  3. **if**

$$\begin{array}{c} AggDBTag^{\prime }==AggDBTag \end{array}$$ (26)

    **then**

      The private verification is positive, i.e. 1.

    **else**

      The private verification is negative i.e. 0.

    **end**

Different from that of STagPriVer, in a BTagPriVer process, multiple tags, {*DBTag*_*i*_}, are verified in a single verification operation. In such a verification, three aggregated data items are freshly computed based on {*En*_*DB*_*i*_}, {*IDTag*_*i*_} and {*DBTag*_*i*_}, respectively. These aggregated data items are *AggEn*_*DB* (an aggregated data value of C data blocks, {*En*_*DB*_*i*_}, computed using EQ (14)), *AggIDTag* (an aggregated identifier tag value of C *IDTags*, {*IDTag*_*i*_}, are computed using EQ (16)), and *AggDBTag* (an aggregated data block tag value, *AggDBTag*, of the set {*DBTag*_*i*_} using EQ (17)), where 0 ≤ *i* ≤ *C*−1, *C* is the number of tags being selected randomly for this verification and *C* ≤ *d*, where *d* is the total number of data blocks. *AggEn*_*DB* and *AggDBTag* are calculated based on the respective items, i.e. {*En*_*DB*_*i*_} and {*DBTag*_*i*_}, by the provider, while *AggIDTag* is computed based on the secret items, i.e. {*IDTag*_*i*_}, by the user. Based on *AggEn*_*DB* and EQ (15), *AggEn*_*DBTag* is computed. Using the computed *AggIDTag* and *AggEn*_*DBTag* and EQ (18), a fresh *AggDBTag*′ is computed, and then compare it with the one retrieved, *AggDBTag*. If the two values are equal, then the integrity of the file is preserved. This verification operation is summarised in the BTagPriVer algorithm (Algorithm 5).

#### File integrity public verification

The public verification of the integrity of a data file refers to the verification of the integrity of a data file by a third party on behalf of the owner of the data file. This is also referred to as tag public verification. Different from the tag private verification method described above, a tag public verification is performed by verifying a BLS signature that has been signed with the file owner’s private key, *x*, with the corresponding public key, *ppk*. Similar to tag private verifications, tag public verifications can also be performed on per tag basis, in which case, it is called Single Tag Public Verification (STagPubVer), or in an aggregated manner, in which case, it is called Batch Tag Public Verification (BTagPubVer).

**Algorithm 6**: STagPubVer

**Input**: *En*_*IDTag_i_*, *En*_*DB_i_*, *DBTag_i_*, *DBTagTag_i_*, *ppk*, *ppk_En_*

**Output**: 0/1

  1. Compute *DataTag*_*i*_ by applying *En*_*DB*_*i*_ to EQ (9).

  2. Compute *En*_*DataTag*_*i*_ by applying *DataTag*_*i*_ and *ppk*_*En*_ to Eq (3).

  3. Compute *En*_*DBTag*_*i*_ by applying *DBTag*_*i*_ and *ppk*_*En*_ to Eq (3).

  4. Compute $En\_DBTag_{i}^{\prime }$ by applying *En*_*IDTag*_*i*_, *En*_*DataTag*_*i*_, to EQ (19).

  5. **if**

$$\begin{array}{c} En\_DBTag_{i}^{\prime }==En\_DBTag_{i} \end{array}$$ (27)

    **then**

      The Verification_1 is positive, i.e. 1.

    **else**

      The Verification_1 is negative, i.e. 0.

    **end**

  6. **if** *Verification_1 == 1*

      (a) Compute *DBTagMapValue*_*i*_ using *DBTag*_*i*_ and MappingFunction algorithm (Algorithm 2).

      (b) Apply *En*_*IDTag*_*i*_, *DBTagMapValue*_*i*_, *DBTagTag*_*i*_ and *ppk* to EQ (20) (Verification_2).

      (c) **if** *Verification_2 == 1* **then**

          The public verification is positive, i.e. 1.

        **else**

          The public verification is negative, i.e. 0.

        **end**

    **else**

      The public verification is negative, i.e. 0.

    **end**

A tag public verification involves the use of four data items, i.e. *En*_*IDTag*_*i*_, *En*_*DB*_*i*_, *DBTag*_*i*_ and *DBTagTag*_*i*_, in the STagPubVer case, or four sets of items, i.e. {*En*_*DB*_*i*_}, {*En*_*IDTag*_*i*_}, {*DBTag*_*i*_} and {*DBTagTag*_*i*_}, in the BTagPubVer case, where *En*_*IDTag*_*i*_ is the encrypted form of *IDTag*_*i*_, and *DBTagTag*_*i*_ is the tag of *DBTag*_*i*_.

It should be emphasised that as {*IDTag*_*i*_} are confidential items, so tag public verifications involved the use of encrypted *IDTags*, i.e. {*En*_*IDTag*_*i*_}. The detailed verification process is as follows. *DataTag*_*i*_ is computed by applying *En*_*DB*_*i*_ to *AS*(), while *En*_*DataTag*_*i*_ and *En*_*DBTag*_*i*_ are computed by applying *DataTag*_*i*_ and *DBTag*_*i*_ to Eq (3), and a fresh *En*_*DBTag*, i.e. $En\_DBTag_{i}^{\prime }$, is computed by applying *En*_*IDTag*_*i*_ and *En*_*DataTag*_*i*_ to EQ (19). It then confirms if the freshly computed $En\_DBTag_{i}^{\prime }$ is equal to the encrypted form of the retrieved *DBTag*_*i*_, i.e. *En*_*DBTag*_*i*_. If this verification is positive, it computes *DBTagMapValue*_*i*_ using *DBTag*_*i*_ and *MappingSecretKey*, as shown in Algorithm 2, and then applies *En*_*IDTag*_*i*_, *DBTagMapValue*_*i*_, *DBTagTag*_*i*_ and the public key, *ppk*, to EQ (20). If EQ (20) holds, then the verification is positive or true (1). Otherwise, it is negative or false (0). The algorithm for this verification is detailed in Algorithm 6 (i.e. STagPubVer algorithm). Similar to the case of BTagPriVer, batch tag public verification (BTagPubVer) also allows multiple tags (i.e. {*DBTagTag*_*i*_}) to be verified in a single verification operation. The algorithm for this verification is summarised in Algorithm 7 (i.e. BTagPubVer algorithm).

**Algorithm 7**: BTagPubVer

**Input**: *AggEn*_*DB*, *AggDBTagTag*, {*En*_*IDTag_i_*}, {*DBTag_i_*}, where 0 ≤ *i* ≤ *C* − 1, *ppk*, *ppk_En_*

**Output**: 0/1

  1. Compute *AggEn*_*IDTag* and *AggDBTag* by applying {*En*_*IDTag*_*i*_} and {*DBTag*_*i*_} to EQs (EQ (21)) and (EQ (17)), respectively.

  2. Compute *AggEn*_*DBTag* by applying *AggEn*_*DB* to EQ (15).

  3. Compute *En*_*AggEn*_*DBTag* and *En*_*AggDBTag* by applying *AggEn*_*DBTag*, *AggDBTag* and *ppk*_*En*_ to Eq (3).

  4. Compute a fresh *En*_*AggDBTag*, *En*_*AggDBTag*′, by applying *AggEn*_*IDTag* and *En*_*AggEn*_*DBTag* to EQ (22).

  5. **if**

$$\begin{array}{c} En\_AggDBTag^{\prime }==En\_AggDBTag \end{array}$$ (28)

    **then**

      The Verification_1 is positive, i.e. 1.

    **else**

      The Verification_1 is negative, i.e. 0.

    **end**

  6. **if** *Verification_1 == 1* **then**

      (a) Computes {*DBTagMapValue*_*i*_} using {*DBTag*_*i*_} and MappingFunction algorithm (Algorithm 2).

      (b) Apply {*En*_*IDTag*_*i*_}, {*DBTagMapValue*_*i*_}, *AggDBTagTag* and *ppk* to EQ (24) (Verification 2).

      (c) **if** *Verification_2 == 1* **then**

          The public verification is positive, i.e. 1.

        **else**

          The public verification is negative, i.e. 0.

        **end**

    **else**

      The public verification is negative, i.e. 0.

    **end**

## 5 Correctness and security analysis

In this section, we analyse the correctness and security of the TOD method. The analysis makes use of the security requirements specified in section 2.2.

### 5.1 Correctness

**Theorem 1**: Given a data file and its tags, the verifier can verify the integrity of the data file.

**Proof**: Proving the correctness of the TOD method is equivalent to proving the correctness of equations, Eqs (26), (28) and ((24)). Based on property of the algebraic signature, i.e. *AS*(*DB*_1_) + *AS*(*DB*_2_) = *AS*(*DB*_1_ + *DB*_2_) as indicated in Eq (7), the homomorphic addition property in Paillier as indicated in Eq (5) and the bilinear pairing described in Section 4.3, all the three equations, as verified below, hold.

Eq (26):

*AggDBTag*′ = *AggDBTag*

**Left Side**: *AggDBTag*′

$$\begin{array}{cc} & =AggIDTag+AggEn\_DBTag,\;(\text{based}\;\text{on}\;\text{EQ}(18))\\ & =\sum_{i=0}^{C-1}IDTag_{i}+AS\left(AggEn\_DB\right),\;(\text{based}\;\text{on}\;\text{EQ}(15)\text{and}\;\text{EQ}(16))\\ & =\sum_{i=0}^{C-1}IDTag_{i}+AS\left(\sum_{i=0}^{C-1}En\_DB_{i}\right),\;(\text{based}\;\text{on}\;\text{EQ}(14))\\ & =\sum_{i=0}^{C-1}IDTag_{i}+\sum_{i=0}^{C-1}AS\left(En\_DB_{i}\right),\;(\text{based}\;\text{on}\;\text{EQ}(7))\\ & =\sum_{i=0}^{C-1}IDTag_{i}+\sum_{i=0}^{C-1}DataTag_{i},\;(\text{based}\;\text{on}\;\text{EQ}(9))\\ & =\sum_{i=0}^{C-1}\left[IDTag_{i}+DataTag_{i}\right]\\ & =\sum_{i=0}^{C-1}DBTag_{i},\;(\text{based}\;\text{on}\;\text{EQ}(10))\\ & =AggDBTag,\;(\text{based}\;\text{on}\;\text{EQ}(17)) \end{array}$$

Eq (26) holds.

Eq (28):

*En*_*AggDBTag*′ = *En*_*AggDBTag*

**Left Side**: *En*_*AggDBTag*′

$$\begin{array}{cc} & =AggEn\_IDTag\times En\_AggEn\_DBTag,\;(\text{based}\;\text{on}\;\text{EQ}(22))\\ & =\prod_{i=0}^{C-1}En\_IDTag_{i}\times E\left(AggEn\_DBTag\right),\;(\text{based}\;\text{on}\;\text{EQ}(21)\;\text{and}\;\text{EQ}(3))\\ & =\prod_{i=0}^{C-1}En\_IDTag_{i}\times E\left(AS\left(AggEn\_DB\right)\right),\;(\text{based}\;\text{on}\;\text{EQ}(15))\\ & =\prod_{i=0}^{C-1}En\_IDTag_{i}\times E\left(AS\left(\sum_{i=0}^{C-1}En\_DB_{i}\right)\right),\;(\text{based}\;\text{on}\;\text{EQ}(14))\\ & =\prod_{i=0}^{C-1}En\_IDTag_{i}\times E\left(\sum_{i=0}^{C-1}AS\left(En\_DB_{i}\right)\right),\;(\text{based}\;\text{on}\;\text{EQ}(7))\\ & =\prod_{i=0}^{C-1}En\_IDTag_{i}\times E\left(\sum_{i=0}^{C-1}DataTag_{i}\right),(\text{based}\;\text{on}\;\text{EQ}(9))\\ & =\prod_{i=0}^{C-1}En\_IDTag_{i}\times \prod_{i=0}^{C-1}En\_DataTag_{i},(\text{based}\;\text{on}\;\text{EQ}(5))\\ & =E(\sum_{i=0}^{C-1}\left(IDTag_{i}+DataTag_{i}\right)),\;(\text{based}\;\text{on}\;\text{EQ}(5))\\ & =E(\sum_{i=0}^{C-1}DBTag_{i}),\;(\text{based}\;\text{on}\;\text{EQ}(10))\\ & =E(AggDBTag),\;(\text{based}\;\text{on}\;\text{EQ}(17))\\ & =En\_AggDBTag,\;(\text{based}\;\text{on}\;\text{EQ}(3)) \end{array}$$

Eq (28) holds.

EQ (24):

$$\begin{array}{c} e(AggDBTagTag,g_{2})=e(\prod_{i=0}^{C-1}H\left(En\_IDTag_{i}\right)\times \upsilon^{\sum_{i=0}^{C-1}DBTagMapValue_{i}},ppk) \end{array}$$

**Left Side**: *e*(*AggDBTagTag*, *g*_2_)

**Right Side**:

$e(\prod_{i=0}^{C-1}H\left(En\_IDTag_{i}\right)\times \upsilon^{\sum_{i=0}^{C-1}DBTagMapValue_{i}},ppk)$

$$\begin{array}{cc} & =e(\prod_{i=0}^{C-1}H\left(En\_IDTag_{i}\right)\times \prod_{i=0}^{C-1}\upsilon^{DBTagMapValue_{i}},ppk),\\ & \;\text{based}\;\text{on}\;ppk=g_{2}^{x},\;\text{as}\;\text{described}\;\text{in}\;\text{the}\;\text{BLS-KeyG}\;\text{Algorithm}\\ & \;\text{in}\;\text{section}\;4.3:\\ & =e(\prod_{i=0}^{C-1}\left[H\left(En\_IDTag_{i}\right)\times \upsilon^{DBTagMapValue_{i}}\right],g_{2}^{x}),\\ & \;\text{based}\;\text{on}\;\text{property}\;(\text{P1})\;\text{of}\;\text{the}\;\text{bilinear}\;\text{pairing,}\;\text{as}\;\text{described}\;\text{in}\\ & \;\text{the}\;\text{BLS}\;\text{scheme}\;\text{in}\;\text{section}\;4.3:\\ & =e(\prod_{i=0}^{C-1}{\left[H\left(En\_IDTag_{i}\right)\times \upsilon^{DBTagMapValue_{i}}\right]}^{x},g_{2})\\ & =e(\prod_{i=0}^{C-1}DBTagTag_{i},g_{2}),\;(\text{based}\;\text{on}\;\text{EQ}(11))\\ & =e(AggDBTagTag,g_{2}),\;(\text{based}\;\text{on}\;\text{EQ}(23))\\ & \text{EQ}(24)\;\text{holds}. \end{array}$$

### 5.2 Tag forgery resistance

In this section, we analyse the cost for circumventing private verifiability via tag forgeries. The notations used in this security analysis are summarised in Table 6.

**Table 6:** Notations used in the security analysis.

Notation	Description
*L*_*X*_	Bit-length of X
*BFA*_*X*_	Computational cost on brute force attack on X
*P*_*Z*_	Probability of finding a collision in Z
*m*	Degree of *GF*(2^*m*^)
*PE*	Primitive elements in *GF*(2^*m*^)
*TCSC*	Total number of Cycles per Second per Core
*TCP*	Total number of Cores per Processor
*TPD*	Total number of Processors per Device
*TSY*	Total number of Seconds per Year
*N*_*Y*_	Total Number of all possible combinations of Y, i.e. the space size of *Y*
*ECN*	Estimated Cycle Number per combination check
*DY*	Device-Year

As indicated in our assumptions (see assumptions, A1 and A2), the cryptographic algorithms used are secure and cryptographic keys used are securely generated, distributed and stored, so the analyses in this section assume that the attacks on the tags are mounted by using brute-force attacks. The computational cost for forging a tag using brute-force attacks (hereafter referred to as the *BFA* cost) is measured by in terms of server-years required to forge a tag using *BFA* successfully, i.e. given one server or one device, the number of years it takes for an attack to succeed.

The *BFA* cost can be calculated by using Eq (29), which converts the number of all possible combinations (*N*) into one that is measured in the unit of server-year, and this is done by dividing the value of *N* multiplied by the Estimated Cycle Number (*ECN*) per combination check by the Device-Year (*DY*), i.e.

$$\begin{array}{c} BFA\;\text{Cost}=(N\times ECN)/DY(\text{server-years}) \end{array}$$ (29)

where *DY* is a unit used to measure the performance of a device per year; it is defined as the number of cycles per year a device can execute.

Typically, each instruction execution requires a number of cycles. *DY* can be computed by multiplying the following values: the Total number of Cycles per Second per Core (*TCSC*), the Total number of Cores per Processor (*TCP*), the Total number of Processors per Device (*TPD*) and the Total number of Seconds per Year (*TSY*), i.e.

$$\begin{array}{c} DY=TCSC\times TCP\times TPD\times TSY \end{array}$$ (30)

To give a more detailed idea about the *BFA* cost, we here use two types of devices as examples to calculate the cost in the unit of server-years: device type 1 is a server with four (= 2^2^) processors (4ProcDevice), and device type 2 is a PC with one processor (1ProcDevice). Each processor is assumed to have 16 (= 2^4^) cores, and each core has a speed of 2.6 GHz (= 2^31^). According to Eq (30), the DY for 4ProcDevice is 2^31^ × 2^4^ × 2^2^ × 2^25^ = 2^62^, while the DY for 1ProcDevice is 2^31^ × 2^4^ × 1 × 2^25^ = 2^60^. It should be emphasised that the *BFA* cost decreases as the value of *DY* of the used device increases.

The forgery attack may be performed by using multiple devices. In this case, the attack is a distributed *BFA* attack. The *BFA* with distributed attacks can be estimated by using the following equation, where *NUD* is the number of devices used in the attack.

$$\begin{array}{c} \text{Distributed}\;BFA\;\text{Cost}=BFA/NUD \end{array}$$ (31)

It should be emphasised that while the Distributed *BFA* cost decreases as the number of devices used increases, the monetary cost in mounting the attack will increase too. For example, using one 4ProcDevice for an hour from the AWS Amazon service costs around *£*0.23 [41]. Using the device for one year, the cost would be *£*0.23 × *£*8,760 = *£*2,014.8. Using 100 4ProcDevice for one year would put this cost at about 100 × *£*2,014.8 = *£*201,480 in one year.

The following analysis is based on the assumption that non-distributed *BFA* attacks and 4ProcDevice are used.

*IDTags* are secret and they are kept by the data owners themselves. Depending on whether the PCS provider knows the public parameter value, *m* (the degree of GF(2^m^)), used in *IDTag* generations and how the attack is performed, the *BFA* cost will be different. There are three scenarios:

- IDTag-Scenario-1: The PCS provider tries to guess *IDTag* without knowing *m*.
- IDTag-Scenario-2: The PCS provider tries to guess *IDTag* with the knowledge *m*.
- IDTag-Scenario-3: The PCS provider tries to guess *IDTag* via guessing *RN*, the secret random number, and a primitive element used in its generation.

In IDTag-Scenario-1, the attacker needs to guess the length, as well as the value of the tag. The length of an *IDTag*, i.e. *m* for *GF*(2^*m*^), can be set to different values, e.g. *m* = 8, 16, 32, …, etc. Given *m*, the length of an AS tag is m-bits long, thus there are $2^{L_{AS}}=2^{m}$ possible values of an *IDTag* (and this is also referred to as the space of *IDTag* or AS tags, denoted as *N*_*AS*_). Using Eqs (29) and (30), we can calculate the *BFA* for each of the *m* values, *m*_0_, *m*_1_, …, *m*_*n*_ as $BFA_{m_{0}}$, $BFA_{m_{1}}$,…, and $BFA_{m_{n}}$. Then the total cost for Scenario-1 is the sum of the *BFA* s, i.e.

$$\begin{array}{c} BFA_{IDTag-Scenario-1}=\sum_{i=0}^{n}BFA_{m_{i}} \end{array}$$ (32)

In IDTag-Scenario-2, the PCS provider knows the length of an *IDTag*, i.e. the *m* value in *GF*(2^*m*^), so only need to guess this value of the tag. The *BFA* cost in this case is:

$$\begin{array}{c} BFA_{IDTag-Scenario-2}=BFA_{m_{i}} \end{array}$$ (33)

In IDTag-Scenario-3, the PCS provider tries to guess *IDTag* via guessing its input values, *RN*, the secret random number, and the primitive element used in *IDTag* generation as shown in EQ (8). As shown in EQ (8), *IDTag*_*i*_ is computed by applying the algebraic signature to the concatenation of the PCS user’s ID, *User*_*ID*_, and a random number, *RN*_*i*_, and this random number is unique for each data block. The random number is a secret value, i.e. it is only known to the PCS user (the data owner). As *User*_*ID*_ is not secret, the PCS provider needs to guess *RN*_*i*_ as well as the primitive element chosen by the PCS user. Given *m*, the total number of primitive elements (*N*_*PE*_) in *GF*(2^*m*^) can be computed using the following equation:

$$\begin{array}{c} N_{PE}=\frac{\Phi (2^{m}-1)}{m} \end{array}$$ (34)

where Φ(*n*) is the Totient function [42]. For example, *GF*(2^8^) has 16 primitive elements, *GF*(2^16^) has 2048 ≃ 2^11^ primitive elements and *GF*(2^32^) has 67108864 ≃ 2^26^ primitive elements, etc.

Under the assumption that the PCS provider knows the degree of *GF*(2^*m*^) (the weakest link principle), the *BFA*_*IDTag*_ can be calculated using Eq (29), where, *N* = *N*_*RN*_ × *N*_*PE*_, *N*_*RN*_ is the range size of the random number, i.e. $N_{RN}=2^{L_{RN}}$, where *L*_*RN*_ is the bit-length of the random number and *N*_*PE*_ is the total number of primitive elements of *GF*(2^*m*^). *N*_*PE*_ can be computed by using Eq (34).

Based on the above analysis and the weakest link principle, we denote the lowest cost of the three scenarios as the *BFA* cost for *IDTags*, i.e.

$$BFA_{IDTag}=min(BFA_{IDTag\text{0}Scenario\text{0}1},BFA_{IDTag\text{0}Scenario\text{0}2},BFA_{IDTag\text{0}Scenario\text{0}3})$$ (35)

As cost for IDTag-Scenario-1 is the most expensive scenario, so Eq (35) can be written as:

$$BFA_{IDTag}=min(BFA_{IDTag\text{0}Scenario\text{0}2},BFA_{IDTag\text{0}Scenario\text{0}3})\propto min(2^{m},2^{L_{RN}}\times N_{PE})$$ (36)

**Fig 4:**
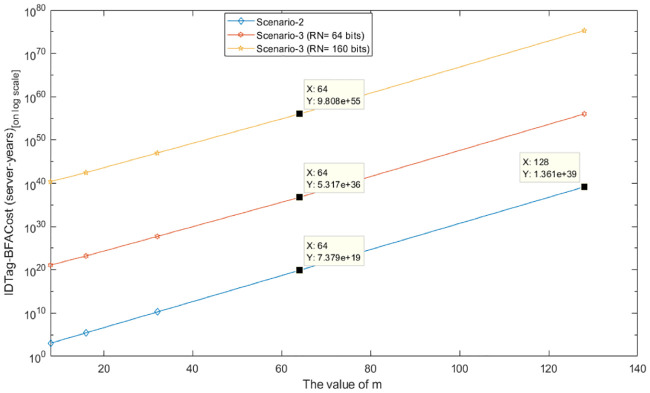
Costs of brute-force attack on *IDTag* versus the value of *m*.

Fig 4, plotted based on Eqs (29) and (36), shows the costs of two scenarios, i.e. Scenario-2 and Scenario-3, for forging *IDTag* vs the value of *m* using *L*_*RN*_ = 64 bits and 160 bits. Based on the figure, we can see that, given other parameter values fixed, *BFA* is determined by *N* which is, in turn, dependent on the length of binary value concerned. This means that IDTag-Scenario-2 is dependent on *m*, the length of *IDTag*, and IDTag-Scenario-3 is dependent on *L*_*RN*_, the length of *RN*, addition to *m*. The cost of Scenario-2 increases as the value of *m* increases. For example, the cost for IDTag-scenario-2 increases from 7.379 × 10^19^ to 1.361 × 10^39^, when the value of *m* increases from 64 to 128. The case for IDTag-scenario-3 is similar as *L*_*RN*_ and *m* increase. For example, given *m* = 64, the cost of scenario-3 increases from 5.317 × 10^36^ to 9.808 × 10^55^, where the bit-length of *RN* increases from 64 to 160. Furthermore, the figure shows that using the scenario-2 can incur the minimum cost compared with the scenario-3, where the bit-length of *RN* is not shorter than *m*. As IDTag-scenario-2 can produce the lower cost,

$$\begin{array}{c} BFA_{IDTag}=BFA_{IDTag-Scenario-2} \end{array}$$ (37)

Therefore, from the results in Fig 4, it can be seen that, to resist *BFA* attack on *IDTags* with the cost more 7.379 × 10^19^ server-years, the *m* value should be more than 64 bits, e.g. 128 bits or more should be chosen for *m*.

As shown in EQ (9), *DataTag*_*i*_ is computed by applying the algebraic signature to the ciphertext of the data block, *En*_*DB*_*i*_, for which the tags are used to protect. As *En*_*DB*_*i*_ is public, so as long as the PCS provider knows the length of *DataTag*_*i*_ and *IDTag*_*i*_ is compromised, it can compute the tag. In other words, the minimum *BFA* cost for *DataTags* is zero, i.e. *BFA*_*DataTag*_ = 0.

Based on EQ (10), *DBTag* is the numerical sum of the corresponding *IDTag* and *DataTag*, i.e. *DBTag*_*i*_ = *IDTag*_*i*_ + *DataTag*_*i*_. As, to the PCS provider, *DataTag*_*i*_ is a known value, if *IDTag*_*i*_ is compromised, then *DBTag*_*i*_ will be compromised. In other words, the *BFA* cost for a *DBTag* is identical to that of an *IDTag*, i.e. *BFA*_*DBTag*_ = *BFA*_*IDTag*_.

*DBTagTag* is a BLS tag. As shows in EQ (11), the generation of *DBTagTag*_*i*_ for a data block, *En*_*DB*_*i*_, involves the use of four items, the hash value of the encrypted *IDTag*_*i*_, i.e. *H*(*En*_*IDTag*_*i*_), a public random element, i.e. *υ*, a map value of *DBTag*_*i*_, i.e. *DBTagMapValue*_*i*_, and the user’s BLS private key, *x*. Among these items, three of them are, or involve the use of, secrets, and these are *IDTag*_*i*_, a mapping key, *MappingSecretKey*, used in *DBTagMapValue*_*i*_ generation, and the BLS private key, *x*. There are three possible ways in which the PCS provider may forge a *DBTagTag*. These scenarios are:

- DBTagTag-Scenario-1: The PCS provider tries to guess the three secrets, i.e. *IDTag*_*i*_, *MappingSecretKey*, and the BLS private key, *x*.
- DBTagTag-Scenario-2: The PCS provider tries to find a collision in *H*(*En*_*IDTag*) (rather than guessing the *IDTag*_*i*_), and guess *MappingSecretKey*, and *x*.
- DBTagTag-Scenario-3: The PCS provider tries to find a collision in *DBTagTag*_*i*_.

In DBTagTag-Scenario-1, the PCS provider needs to brute-force attack on the three secret items, *IDTag*_*i*_, *MappingSecretKey*, and *x*, to successfully forge *DBTagTag*_*i*_. The *BFA* cost on *IDTag*_*i*_, i.e. *BFA*_*IDTag*_, has been devised above and is expressed in Eq (37). *MappingSecretKey* is a randomly selected value with the length of *L*_*MappingSecretKey*_ bits, its range space is *N*_*MappingSecretKey*_, which is equals $2^{L_{MappingSecretKey}}$. With regard to *x*, the BLS private key, it is randomly chosen from *Z*_*p*_. Given a prime *p*, *Z*_*p*_ = {0, 1, 2, …, *p*−1}, the total number of elements in *Z*_*p*_ is (*p*−1), i.e. *N*_*x*_ = (*p*−1). The cost for DBTagTag-Scenario-1, i.e. *BFA*_*DBTagTag*-*Scenario*- 1_, can be calculated using Eq (29), where $N=N_{IDTag}\times N_{MappingSecretKey}\times N_{x}=2^{m}\times 2^{L_{MappingSecretKey}}\times \left(p-1\right))$, where *m* is the length of the tag. *BFA*_*DBTagTag*-*Scenario*- 1_ can be expressed as follows:

$$\begin{array}{cc} BFA_{DBTagTag-Scenario-1}\propto \left[2^{m}\times N_{MappingSecretKey}\times N_{x}\right] & \\ =2^{m}\times 2^{L_{MappingSecretKey}}\times \left(p-1\right) \end{array}$$ (38)

With regard to the cost in DBTagTag-Scenario-2, the difference between this scenario and DBTagTag-Scenario-1 is that, in this scenario, *H*(*En*_*IDTag*_*i*_) is guessed via finding collisions, rather than the brute forace attack on *IDTag*_*i*_, i.e. finding *H*(*En*_*IDTag*_*j*_) for a different *En*_*IDTag* (*En*_*IDTag*_*j*_) that is diffrent from *En*_*IDTag*_*i*_, but *H*(*En*_*IDTag*_*j*_) = *H*(*En*_*IDTag*_*i*_). *H*(*En*_*IDTag*_*i*_) is an element in *G*_1_, its length is $L_{E_{G_{1}}}$, and the average number of trials for finding a collision is $2^{\left(L_{E_{G_{1}}}/2\right)}$. So, we now have:

$$\begin{array}{c} BFA_{DBTagTag-Scenario-2}\propto [2^{\left(L_{E_{G_{1}}}/2\right)}\times 2^{L_{MappingSecretKey}}\\ \times (p-1)] \end{array}$$ (39)

In DBTagTag-Scenario-3, the PCS provider tries to guess *DBTagTag* via finding a collision, i.e. finding *DBTagTag*_*j*_ for a different data block, *En*_*DB*_*j*_, that is different from *En*_*DB*_*i*_, but *DBTagTag*_*j*_ = *DBTagTag*_*i*_. *DBTagTag*_*i*_ is an element in *G*_1_, its length is $L_{E_{G_{1}}}$. So, the cost for DBTagTag-Scenario-3 is:

$$\begin{array}{c} BFA_{DBTagTag-Scenario-3}\propto 2^{\left(L_{E_{G_{1}}}/2\right)} \end{array}$$ (40)

Considering the costs estimated for the three scenarios and the weakest link principle, we have the *BFA* cost for *DBTagTag*, i.e. *BFA*_*DBTagTag*_, as shown in Eq (41).

$$\begin{array}{cc} BFA_{DBTagTag}=min(BFA_{DBTagTag-Scenario-1}, & \\ BFA_{DBTagTag-Scenario-2}, & \\ BFA_{DBTagTag-Scenario-3}) \end{array}$$

$$\begin{array}{cc} \propto min(2^{m}\times 2^{L_{MappingSecretKey}}\times \left(p-1\right),2^{\left(L_{E_{G1}}/2\right)} & \\ \times 2^{L_{MappingSecretKey}\times \left(p-1\right)},2^{\left(L_{E_{G1}}/2\right)}) \end{array}$$ (41)

**Fig 5:**
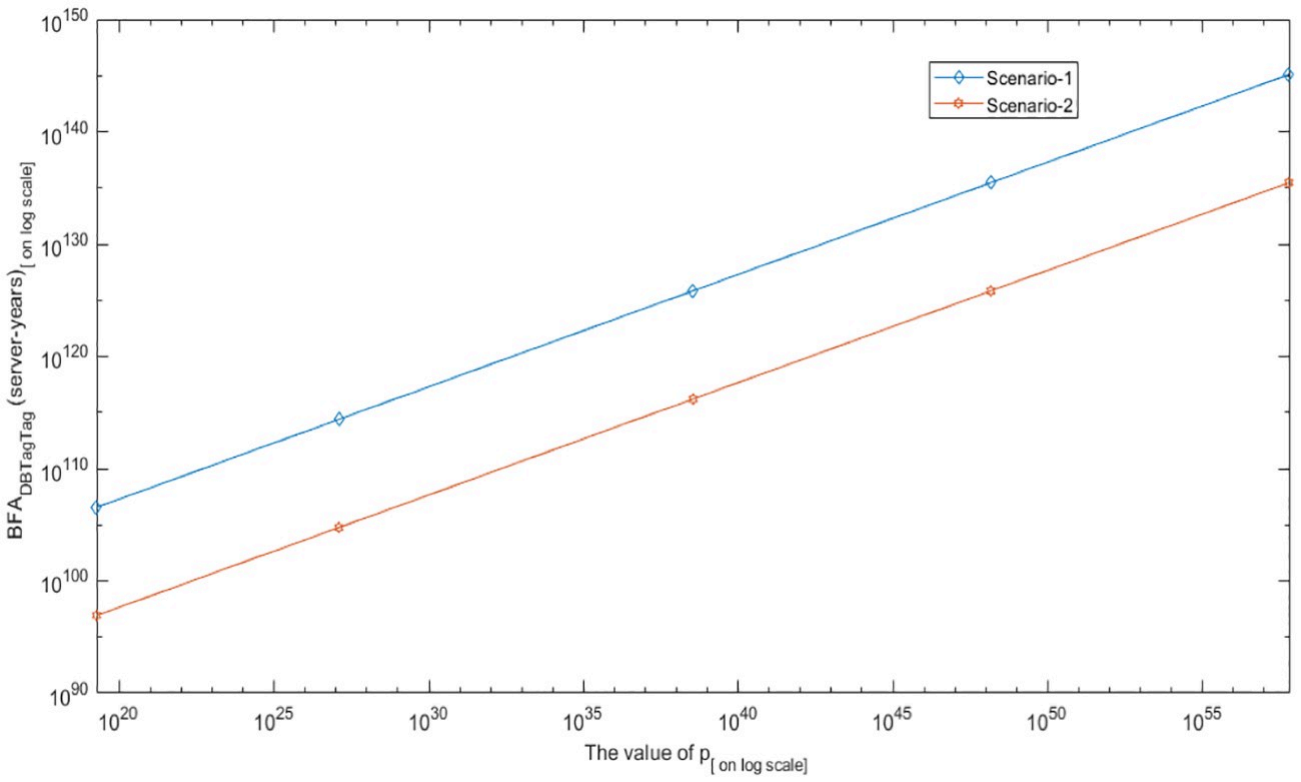
*BFA*_*DBTagTag*_ cost: Scenarios-1 vs Scenarios-2 (*GF*(2^128^), $L_{E_{G_{1}}}=192$ bits and *L*_*MappingSecretKey*_ = 160 bits).

**Fig 6:**
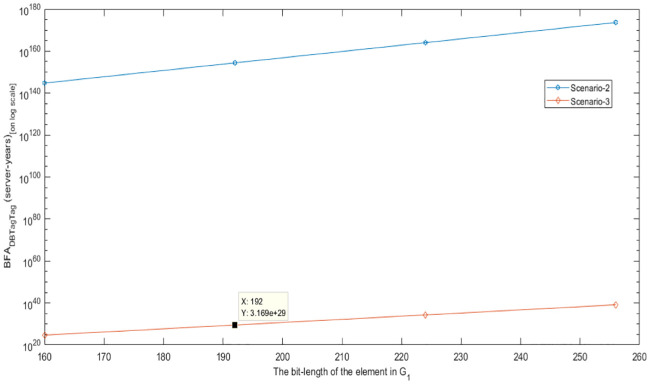
*BFA*_*DBTagTag*_ cost: Scenarios-2 vs Scenarios-3 (*L*_*MappingSecretKey*_ = 160 bits).

Fig 5, plotted based on Eqs (38) and (39), shows the costs of two scenarios, i.e. Scenario-1 and Scenario-2, for forging *DBTagTag* vs the value of *p* using *GF*(2^128^), $L_{E_{G_{1}}}=192$ bits, and *L*_*MappingSecretKey*_ = 160 bits. The two costs increase as the value of *p* increases. The figure shows that using DBTagTag-scenarios-2, the *BFA* cost is the lowest cost, where $L_{E_{G_{1}}}/2$ is shorter than *m*. On the other hand, Fig 6, plotted based on Eqs (39) and (40), shows the costs of two scenarios for *DBTagTag*, i.e. Scenario-2 and Scenario-3, for forging *DBTagTag* vs the bit-length of the element in *G*_1_, $L_{E_{G_{1}}}$, using the value of *p* 2^160^. The two costs increase as the value of $L_{E_{G_{1}}}$ increases. The two figures show that, by finding collisions, i.e. DBTagTag-scenarios-3, the *BFA* cost is the lowest cost. From the results in Fig 6, it can be seen that, to resist *BFA* attack on *DBTagTags* using Scenario-3 with the cost more 3.169 × 10^29^ server-years, $L_{E_{G_{1}}}$ should be more 192 bits long, This means that, in this case, the length of an *DBTagTag* should be longer than 192-bits, e.g. 256 bits.

As indicated in Algorithms 4 and 5, private verifications involve the use of three data items, *En*_*DB*_*i*_, *IDTag*_*i*_ and *DBTag*_*i*_, in the case of single tag verification (the STagPriVer case), or three sets of items, i.e. {*En*_*DB*_*i*_}, {*IDTag*_*i*_} and {*DBTag*_*i*_}, in the case of batch tag verification (the BTagPriVer case). Among the three sets of tags, only {*IDTag*_*i*_} are secrets. The computational cost for circumvent private verification is thus dependent on how many *IDTags* are used in a file integrity verification, how the tags are chosen and how hard it is to compromise each.

In the STagPriVer case, only a single *IDTag* is used per verification and it is chosen randomly. The probability for choosing the right *IDTag* is dependent on the number of such tags that are generated for the file. Assuming that there are *d* sets of tags generated for a file, then the probability for selecting the right IDTag is $Ps=\frac{1}{d}$ [43]. The Average Number of Trials attempted (*ANT*) before selecting the right *IDTag* can computed by the following equation: $ANT=\frac{1-Ps}{Ps}$ [44]. Taking into account that *BFA*_*IDTag*_ ∝ 2^*m*^, the cost for circumvent STagPriVer is:

$$\begin{array}{c} BFA_{STagPriVer}\propto ANT\times 2^{m} \end{array}$$ (42)

Obviously, the higher the value of *d*, the higher the cost of the attack for given values of *m*.

**Fig 7:**
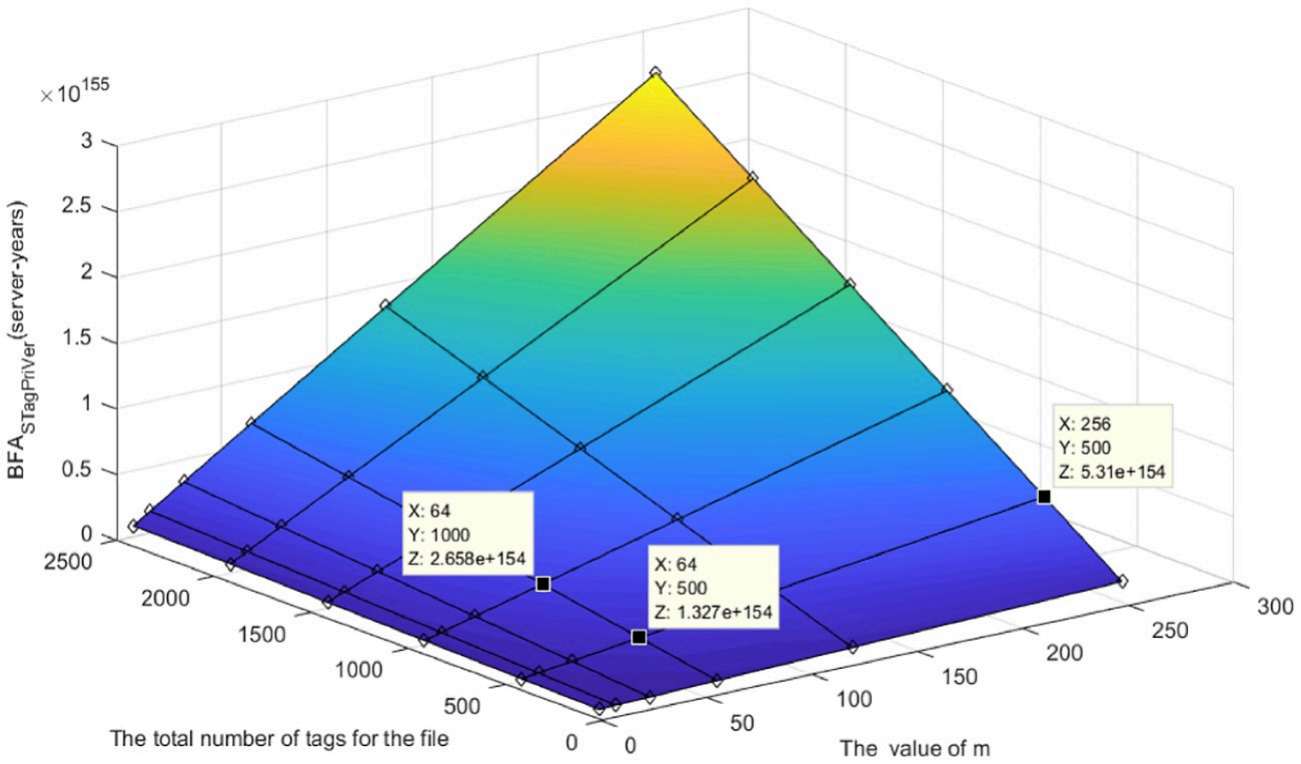
*BFA*_*STagPriVer*_ vs the length of tag (*m*) and the total number of *IDTags* for a data file (*d*).

Fig 7, plotted based on Eqs (29) and (42), shows the effects of the total number of *IDTags*, *d*, that are generated for a data file and the bit-length of the tag, *m*, on *BFA*_*STagPriVer*_. By increasing the number of *IDTags* generated per file or the length of the tag, the *BFA* cost increases. This is because the more the *IDTags* that are generated per file, *d*, the lower the chance a correct set of *IDTags* will be selected, and the longer the bit-length of the tags, the more possible combination number of tags. For example, given *m* = 64, the *BFA*_*STagPriVer*_ increases from 1.327 × 10^154^ to 2.568 × 10^154^ as the value of *d* increases from 500 to 1000. Furthermore, given *d* = 500, the *BFA*_*BTagPriVer*_ increases from 1.327 × 10^154^ to 5.31 × 10^154^ as the value of *m* increases from 64 to 256.

With regard to the BTagPriVer case, there are two ways to circumvent the verification process, one is via finding the values of individual *IDTags* that are used in a BTagPriVer verification, and the other is via finding the value of the *AggDBTag* via collisions. In the former case, in addition to the need for finding the values of the set of *IDTags* used in a verification, one also need to find the right set of *IDTags*. Given that the bit-lengths of an *IDTag* and an *AggDBTag* are identical, and IDTag-scenario-2 is the minimum cost and proportional to 2^m^, the minimum *BFA* cost for BTagPriVer is equal to the *BFA* cost for finding a collision in *AggDBTag*, which is proportional to 2^*m*^.

Based on the above analysis, we can remark that the security level of private verifiability is determined by the security of *IDTags*, which is, in turn, determined by the bit-length of the *IDTags*. This means that the bit-length of the tag should be sufficiently long, e.g. 256-bits.

As tag public verification are based on tags that are all public (i.e. that all can be accessible by the third parties), attempts to circumvent public verifications can only be performed via finding collisions in the tags. The analysis with regard to tag resistance to collision is given in the next section.

### 5.3 Tag collision resistance

In this section, we analyse the level of tag resistance to collisions and this is done by estimating the probabilities for having collisions. A collision refers to two (or more) identical tags that are generated for different data blocks of different PCS users (Collision Type 1, or CT1) or for different data blocks of the same PCS user (Collision Type 2 or CT2). If there are collisions, then it is possible for the PCS provider to use tags that are generated for one data block (of the same user or a different user) for the verification of another data block. Such an attempt is also called a replace attack. As Collision Type 2 is a subset of Collision Type 1, in the following, our analysis will be based on Collision Type 1. Tag collisions may be exploited by third parties to circumvent public verifications.

We use *P*_*Z*_ to denote the probability for finding a collision in *Z* in the worse-case scenario (i.e. the scenario with the highest probability), where *Z* can be any of these tags, *En*_*DB*, *En*_*IDTag*, *DBTag*, *DBTagTag*. For each such tag, there are two ways of finding a collision, one is via finding collisions in their respective inputs (the resulting probability is denoted as *Input*-*P*_*Z*_), and the other is via finding collisions in the *Z* value itself (this probability is denoted as *Output*-*P*_*Z*_). What we are interested in is the factors that influence the values of these probabilities, and the probability, *P*_*Z*_, for the most likely avenue, where

$$\begin{array}{c} P_{Z}=max\left(Input-P_{Z},Output-P_{Z}\right) \end{array}$$ (43)

#### Probability for finding collisions in *En*_*DB* (Z = *En*_*DB*)

The generation of a ciphertext data block, *En*_*DB*_*i*_, involves the use of the LiSHE scheme and a secret key, *sk*. Each PCS user chooses his/her own secret key independently. Encrypting the same plaintext data block with a different secret key will generate a different ciphertext data block. Given two identical plaintext blocks and assuming that the bit-length of the secret key, *sk*, is *L*_*sk*_ and that the secret key are selected uniformly and randomly, the probability for two or more users to select the same secret key thus generating the same ciphertext data block can be estimated based on the generalised birthday problem [45] and can be calculated as follows:

$$\begin{array}{cc} Input-P_{En\_DB}=1-e^{\left(-N_{U}\times \left(N_{U}-1\right)\right)/\left(2\times N_{sk}\right)} & \\ \approx 1-e^{\left(-{\left(N_{U}\right)}^{2}\right)/\left(2\times N_{sk}\right)} \end{array}$$ (44)

where *N*_*U*_ is the total number of users managed by the PCS provider and *N*_*sk*_ is the space (i.e. range size) of *sk*.

With regard to the value of *N*_*sk*_, this can be calculated as follows. As mentioned in Section 5.2, the secret key, *sk*, in the LiSHE scheme, consists of two values, *s* and *q*. So the number of possible combinations (i.e. *N*_*sk*_) is *N*_*s*_ × *N*_*q*_, where *N*_*s*_ is the space of *s* and *N*_*q*_ is the space of *q*.

For a given block length, *L*_*En*_*DB*_, of *En*_*DB*, there are *N*_*En*_*DB*_ possible values of *En*_*DB*, where $N_{En\_DB}=2^{L_{En\_DB}}$. If the total number of encrypted data blocks managed by the PCS provider is *N*_*DB*_, and it is larger than *N*_*En*_*DB*_, the space of *En*_*DBs*, then it is possible that there are two or more *En*_*DBs* with the same value (i.e. *En*_*DB* collisions) regardless of their inputs. Also, as *N*_*DB*_ ≫ 1, we have this probability as follows:

$$\begin{array}{cc} Output-P_{En\_DB}=1-e^{\left(-N_{DB}\times \left(N_{DB}-1\right)\right)/\left(2\times N_{En\_DB}\right)} & \\ \approx 1-e^{\left(-{\left(N_{DB}\right)}^{2}\right)/\left(2\times N_{En\_DB}\right)} \end{array}$$ (45)

Based on Eqs (44) and (45), we have the worse-case probability of *En*_*DB* collisions as shown in Eq (46). It is reasonable to assume that *N*_*DB*_ ≫ *N*_*U*_, as each user typically has multiple files and each file is typically divided into multiple data blocks.

$$\begin{array}{c} P_{En\_DB}=max(1-e^{\left(-{\left(N_{U}\right)}^{2}\right)/\left(2\times N_{sk}\right)},\\ 1-e^{\left(-{\left(N_{DB}\right)}^{2}\right)/\left(2\times N_{En\_DB}\right)}) \end{array}$$ (46)

#### Probability for finding collisions in *DataTag* (Z = *DataTag*)

*DataTags* are used for computing *DBTags*, as indicated in EQ (10). *DataTags* are computed by applying the algebraic signature to encrypted data blocks, *En*_*DBs*, using the primitive elements chosen by the PCS user. The probability of having collision on these inputs is, *Input*-*P*_*DataTag*_ = *P*_*En*_*DB*_ × *P*_*PE*_ = *max*(1 − *e*^(−(*N*_*U*_)^2^)/(2 × *N*_*sk*_)^, 1 − *e*^(−(*N*_*DB*_)^2^)/(2 × *N*_*En*_*DB*_)^ × (1 − *e*^(−(*N*_*U*_)^2^)/(2 × *N*_*PE*_)^).

The probability of finding a collision in *DataTags* is via finding collisions in the values of these tags is, *Output*-*P*_*DataTag*_ ≈ (1 − *e*^(−(*N*_*DB*_)^2^)/(2 × *N*_*AS*_))^. AS an easier way to find a collision in *DataTags* is via finding collisions in the values of these tags, rather than via finding collisions in their input values, so we have,

$$\begin{array}{cc} P_{DataTag} & =max(Input-P_{DataTag},Output-P_{DataTag})\\ & =Output-P_{DataTag})\approx \left(1-e^{\left(-{\left(N_{DB}\right)}^{2}\right)/\left(2\times N_{AS}\right)}\right) \end{array}$$ (47)

#### Probability for finding collisions in *DBTag* (Z = *DBTag*)

For the similar reasons as stated for *DataTags* above, the worse-case probability for finding a collision in *DBTags* is as follows:

$$\begin{array}{cc} P_{DBTag}=Output-P_{DBTag}=Output-P_{DataTag} & \\ \approx 1-e^{\left(-{\left(N_{DB}\right)}^{2}\right)/\left(2\times N_{AS}\right)} \end{array}$$ (48)

#### Probability for finding collisions in *En*_*IDTag* (Z = *En*_*IDTag*)

*En*_*IDTags* are produced based on *IDTags* and using Paillier scheme, so an easier way to find a collision in *En*_*IDTags* is via finding collisions in the values of these *En*_*IDTags*, rather than via finding collisions in their input values. The probability for finding collisions in *En*_*IDTags* is depended on the number of tags that are generated, *N*_*DB*_, and the total number of possible values an *En*_*IDTag* may be set to, *N*_*Paillier*_. Paillier scheme can produce a ciphertext ∈ $Z_{n^{2}}^{*}$, so *N*_*Paillier*_ = *n*^2^ − 1, as shown in Eq (3). So, the probability is:

$$\begin{array}{cc} P_{En\_IDTag} & =1-e^{\left(-{\left(N_{DB}\right)}^{2}\right)/\left(2\times N_{Paillier}\right)}\\ & =1-e^{\left(-{\left(N_{DB}\right)}^{2}\right)/\left(2\times \left(n^{2}-1\right)\right)} \end{array}$$ (49)

#### Probability for finding collisions in *DBTagTag* (Z = *DBTagTag*)

*DBTagTags* are BLS tags. Similar to the analysis of AS tags (i.e. *DBTags*), there are also two ways of generating two identical BLS tags for two different data blocks. One is by finding collisions in the inputs of the tag generation algorithm (the probability is denoted as *Input*-*P*_*DBTagTag*_), and the other is by finding collisions in *DBTagTag* values (the probability is denoted as *Output*-*P*_*DBTagTag*_).

The generation of *DBTagTag* involves the use of the following items (see EQ (11)): (1) *H*(*En*_*IDTag*) which is an element in *G*_1_, (2) *DBTagMapValue* which is a hash value of *DBTag* using MappingFunction, (3) a random number, *υ*, which is an element in *G*_1_, and (4) a user-dependent private BLS key, *x*. Successfully mounting a replace attack via finding collisions on the input values requires one to find collisions on the values of all the four items. This probability, *Input*-*P*_*DBTagTag*_, is the multiplication of four further probabilities. As *N*_*H*(*En*_*IDTag*)_ = *N*_*DBTagMapValue*_ = *N*_*DB*_ and *N*_*υ*_ = *N*_*x*_ = *N*_*U*_, so the *Input*-*P*_*DBTagTag*_ can be as follows.

$$\begin{array}{c} Input-P_{DBTagTag}=P_{H(En\_IDTag)}\times P_{MappingFunction}\times \\ P_{\upsilon }\times P_{x}\\ =\left(1-e^{\left(-{\left(N_{DB}\right)}^{2}\right)/\left(2\times 2^{L_{E_{G_{1}}}}\right)}\right)\times \left(1-e^{\left(-{\left(N_{DB}\right)}^{2}\right)/\left(2\times p-1\right)}\right)\\ \times \left(1-e^{\left(-{\left(N_{U}\right)}^{2}\right)/\left(2\times 2^{L_{E_{G_{1}}}}\right)}\right)\times \left(1-e^{\left(-{\left(N_{U}\right)}^{2}\right)/\left(2\times p-1\right)}\right) \end{array}$$ (50)

The probability of having a collision in the value of *DBTagTag* is: $Output-P_{DBTagTag}=1-e^{\left(-{\left(N_{DB}\right)}^{2})/(2\times 2^{L_{E_{G_{1}}}}\right)}$, where $L_{E_{G_{1}}}$ is the bit-length of *DBTagTag*. As *Output*-*P*_*DBTagTag*_ produces a bigger value than *Input*-*P*_*DBTagTag*_, so we have,

$$\begin{array}{c} P_{DBTagTag}=max\left(Input-P_{DBTagTag},Output-P_{DBTagTag}\right)\\ =Output-P_{DBTagTag}\approx 1-e^{\left(-{\left(N_{DB}\right)}^{2}\right)/\left(2\times 2^{L_{E_{G_{1}}}}\right)} \end{array}$$ (51)

#### Probability for circumventing public verification via collisions

To successfully mount a replace attack on the public verification of a data block, *En*_*DB*_*i*_, the PCS provider needs to find another ciphertext data block, *En*_*DB*_*j*_, where *En*_*DB*_*j*_ ≠ *En*_*DB*_*i*_, that is tagged with *En*_*IDTag*_*j*_, *DBTag*_*j*_ and *DBTagTag*_*j*_, but the tags satisfy the following condition, i.e. *En*_*IDTag*_*j*_ = *En*_*IDTag*_*i*_, *DBTag*_*j*_ = *DBTag*_*i*_ and *DBTagTag*_*j*_ = *DBTagTag*_*i*_. The probability for satisfying this condition can be expressed as *P*_*PubVer*_ and it is:

$$\begin{array}{c} P_{PubVer}=P_{En\_IDTag}\times P_{DBTag}\times P_{DBTagTag} \end{array}$$

$$=(1-e^{\left(-{\left(N_{DB}\right)}^{2})/(2\times N_{Paillier}\right)})\times (1-e^{\left(-{\left(N_{DB}\right)}^{2})/(2\times N_{AS}\right)})\times (1-e^{\left(-{\left(N_{DB}\right)}^{2})/(2\times N_{E_{G_{1}}}\right)})$$ (52)

It can be seen from the equation that the collision probability is dependent on four parameter values. The first is the total number of data blocks (*N*_*DB*_) manged by the PCS provider, and this number is, in turn, dependent on the total number of the users (*N*_*U*_) served by the system and the average number of data blocks uploaded per user ($N_{A_{DB}}$), i.e. $N_{DB}=N_{U}\times N_{A_{DB}}$. The second is the length of AS tag, i.e. *m*, where *N*_*AS*_ = 2^m^. The third is the length of $E_{G_{1}}$, where, $N_{E_{G_{1}}}=2^{L_{E_{G_{1}}}}$. The fourth is the total possible number of the ciphertexts can be produced using Paillier scheme, *N*_*Paillier*_.

**Fig 8:**
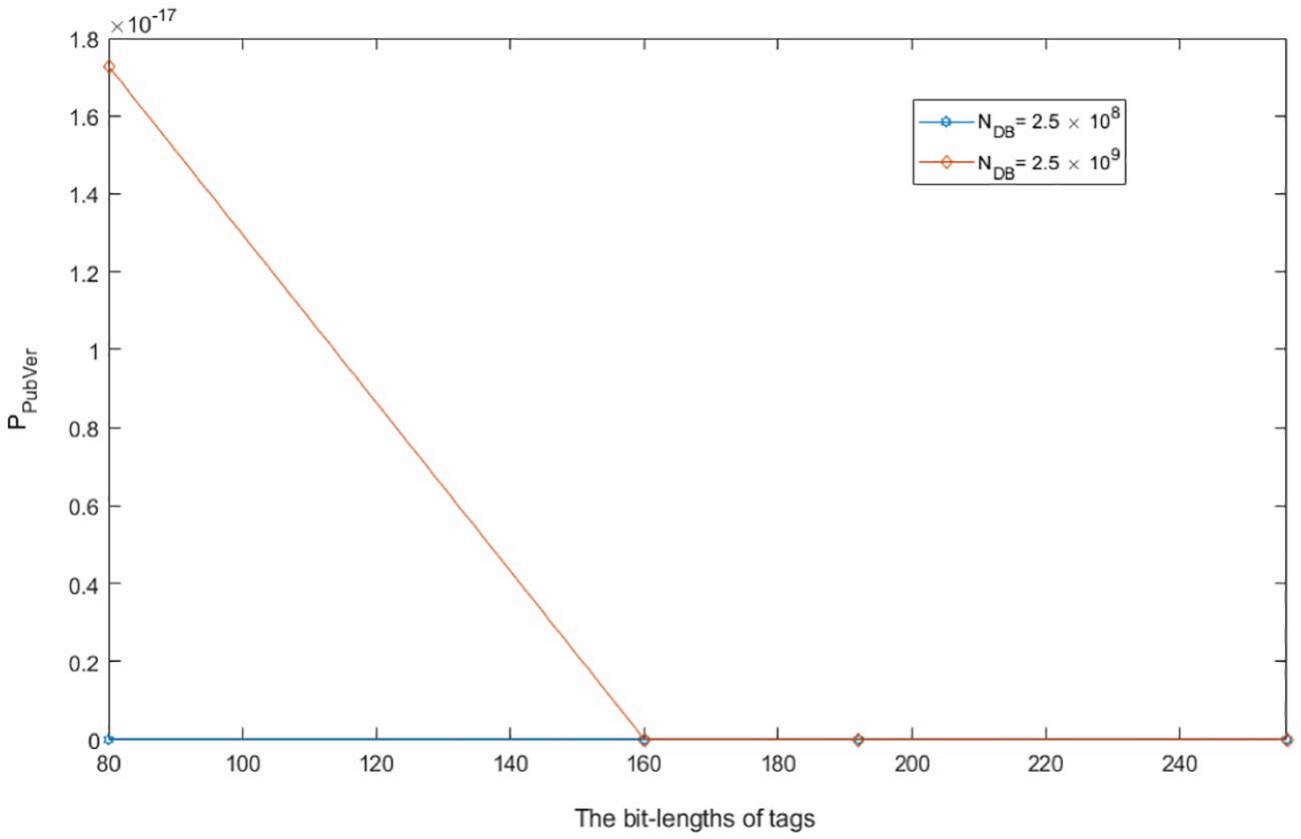
*P*_*PubVer*_ vs. the bit-lengths of *DBTag*, *En_IDTag* and *DBTagTag*.

Fig 8 shows the collision probability vs the bit-length of the tags, under the assumptions that the number of users managed by the PCS provider are respectively, 50,000 and 500,000 (so the *N*_*DB*_ values are respectively 2.5 × 10^8^ and 2.5 × 10^9^ given $N_{A_{DB}}=5,000$). Based on the figures, we can see that, the probability of collision increases as the bit-lengths of the tags decreases and the total number of data blocks, *N*_*DB*_, increases, which is, in turn, dependent on the total number of the user, *N*_*U*_, and the average of data blocks of each PCS user, $N_{A_{DB}}$.

### 5.4 Non-repudiation of tag generation

In this section, we analyse how a repudiation attack may be mounted by a PCS user and the level of efforts required in resisting such an attack.

A dishonest PCS user may repudiate (i.e. falsely deny) the generation of some tags in an attempt such as seeking some benefits from the service provider. In TOD, this can be thwart by using the BLS tags, i.e. *DBTagTags*. As the key used to generate a *DBTagTags* is a BLS private key that is only known to its owner, a PCS user, and the verification key is the corresponding BLS public key, provided that (i) each public key is certified, (ii) that there is a public key certificate revocation system so that any compromised or suspected to have been compromised keys can be revoked promptly, and (iii) that the hash functions used in the tag generations are strong collision resistant [46], it is hard for the PCS user to repudiate the generation of DBTagTags. Conditions (i) and (ii) can be satisfied by implementing proper key management procedures and facilities. In the following, we discuss satisfying condition (iii).

However, if the hash functions used in the *DBTagTags* generation are not strong collision resistant, it is possible for a PCS user to exploit hash value collisions to repudiate the generation of a *DBTagTag*. A user may construct an alternative explanation [47] to argue that a *DBTagTag* is mathematically valid, but she/he has never generated the tag, thus succeeding in repudiating the generation of the tag. The alternative explanation attack is via finding collisions in hash values used in *DBTagTag* generation (the resulting cost is denoted as *BFA* for the Alternative Explanation via finding Collisions in Hash values (*BFA*_*AECH*_)).

Based on EQ (11), two hash values are used in *DBTagTag* generation, i.e. *H(En_IDTag)* and *DBTagMapValue*. *H*(*En*_*IDTag*) is an element in *G*_1_ and its length is $L_{E_{G_{1}}}$, and *DBTagMapValue* is a map value of *DBTag* using MappingFunction, which is ∈*Z*_*p*_. The *BFA*_*AECH*_ can be calculated using Eq (29), where, $N=2^{(L_{E_{G_{1}}})/2}\times \left(p-1\right)/2$.

**Fig 9:**
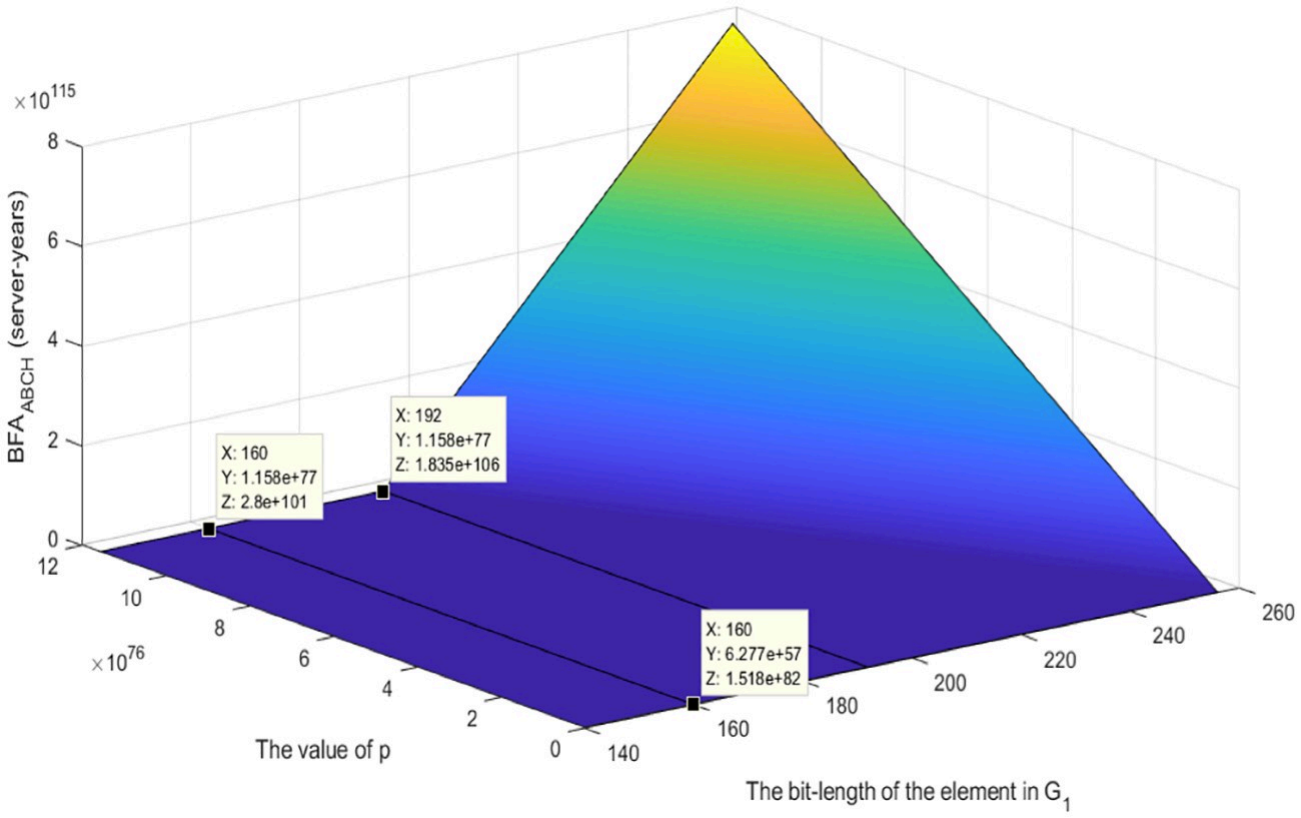
Cost of *BFA*_*AECH*_ vs. the bit-lengths of the element in *G*_1_ and *p*.

Fig 9 shows the cost, *BFA*_*AECH*_, versus the bit-lengths for $E_{G_{1}}$ and the value of *p*. The cost increases as the length of the $L_{E_{G_{1}}}$ and the value of *p* increase. Therefore, for a given the larger the values of $L_{E_{G_{1}}}$ and (*p*−1), the higher cost of *BFA*_*AECH*_.

Furthermore, as *DBTags* are used in generating and verifying *DBTagTags*, *DBTags* can also help to protect against repudiation attacks.

### 5.5 Data confidentiality preservation

Each of the data blocks in a data file is encrypted with a symmetric key, and this key is only known to the PCS user (i.e. the data owner). In addition, tag verifications do not require the access of plaintext data (as shown in Algorithms 4 and 6). Provided that the symmetric key is secure, it is computationally hard for any entities, including the PCS provider and TPA, to access the plaintext data. Also, in TOD, a PCS user does not need to share the symmetric key with other entity, eliminating the need for symmetric key distribution, making the protection of the confidentiality of the data blocks more secure.

**Fig 10:**
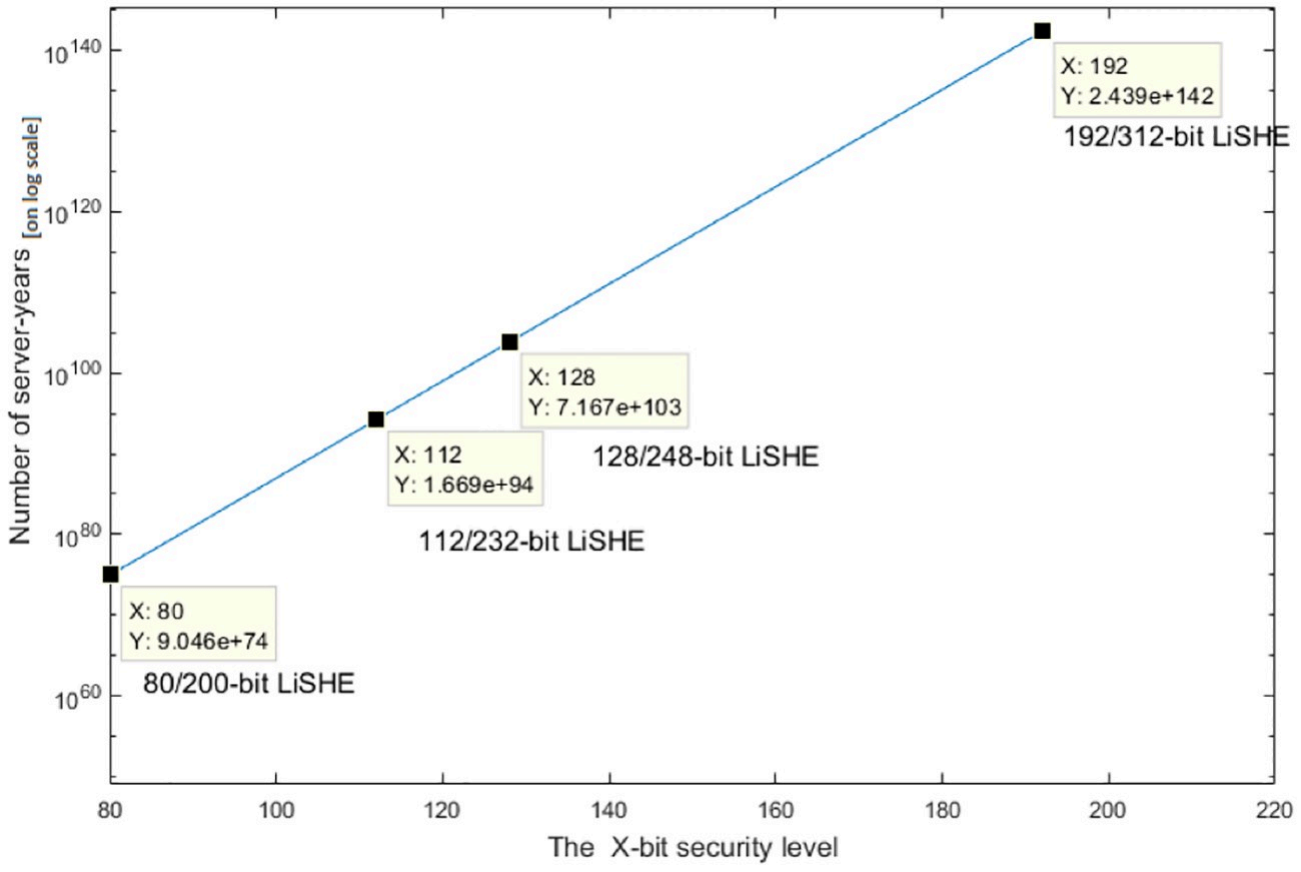
Level of efforts on brute-force attack on a LiSHE key vs. the key length and the key space.

However, one may try to guess the symmetric key using a brute-force method. The LiSHE scheme, the encryption algorithm used to protect the confidentiality of data blocks in TOD, is an existential forgery-secure under known-plaintext attacks as proved in [33]. As mentioned in Section (5.3), the secret key, *sk*, in this scheme, consists of two values *s* and *q*. So, the number of possible combinations (i.e. key space) *N*_*sk*_ is *N*_*s*_ × *N*_*q*_, where *N*_*s*_ is the space of *s* and *N*_*q*_ is the space of *q*. For estimating the cost for brute-force attack on *sk*, Eq (29) can be used. By increasing the length of the key, the key space will increase and so is the cost of cracking it (Fig 10). For example, with an 80 bit security level (*N*_*sk*_ = 2^280^), the cost is 9.046 × 10^74^ server-years.

### 5.6 Unbounded verifiability

Unbounded verifiability is the property for resisting tag replay attacks. In such an attack, the PCS provider dispatches cached aggregated values, *AggEn*_*DB*, *AggDBTag*, and *AggDBTagTag* for batch verification (private or public), rather than freshly compute values. To reduce the success rate of such attacks, in TOD, a random sampling strategy is used when selecting data blocks and their associated tags in each verifications. The selections are made by the verifier, the PCS user in the private verifications and the TPA in the case of public verifications. Each file is divided into *K* data blocks. In each verification, *C* out of *K* blocks are randomly select, where 1 ≼ *C* ≼ *K*. The number of possible combinations in the selection (i.e. selection space) is *K*!/*C*!(*K* − *C*)!, where! is a factorial notation [43]. For example, given a file size of 100 data blocks, the maximum number of possible combinations for randomly selecting *C* = 30 data blocks in the verification is 100!/30!(100 − 30)! = 2.93 × 10^25^. Obviously, the selection space is dependent on the size of a file and the length of each block. The bigger the file size or the smaller the data block, the larger the *K*, which means the bigger the selection space, thus the higher the resistance against the tag replay attacks.

**Fig 11:**
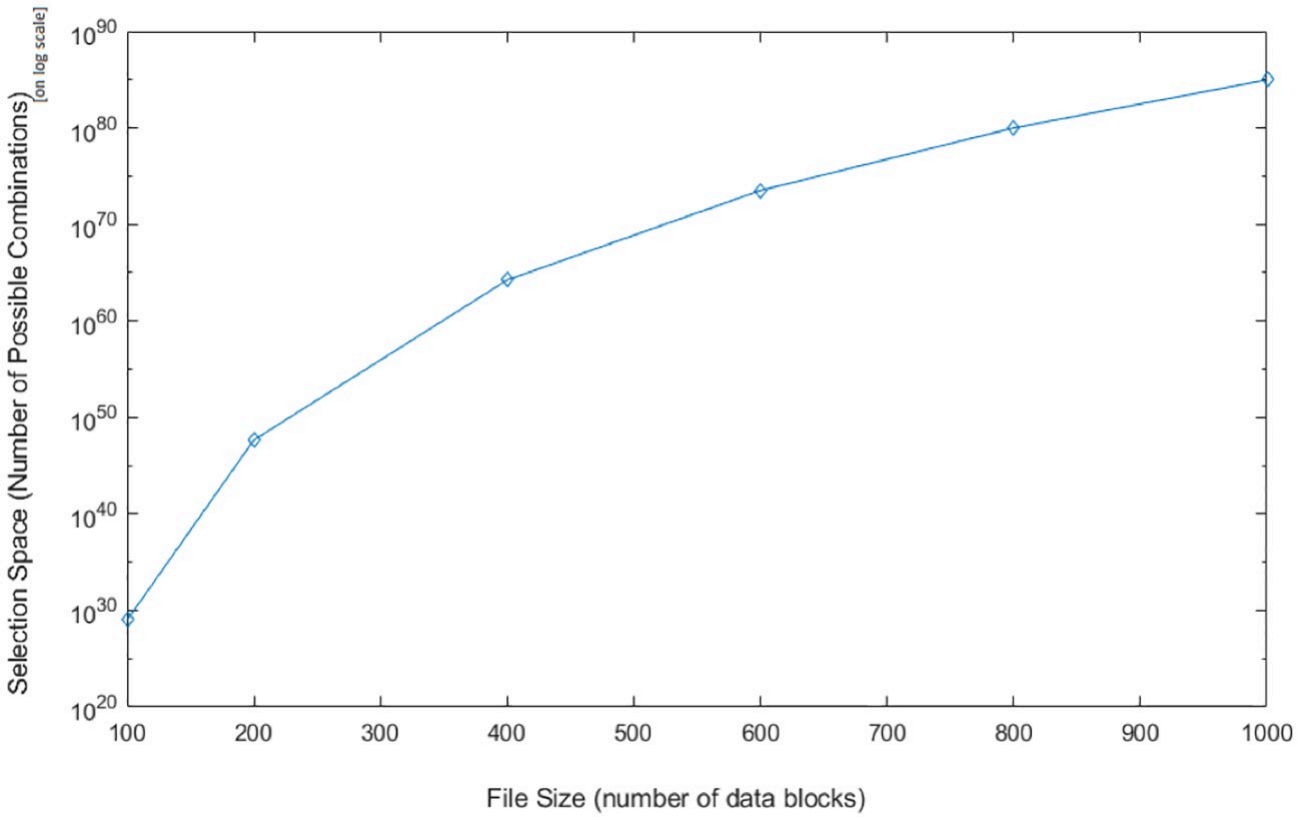
Given C = 50 blocks: Selection space (number of possible combinations) vs. file size (number of data blocks).

Fig 11 shows selection space versus of the file size, assuming C = 50 blocks. It is worth mentioning that C can be a variable, in which case the selection space can further increased.

## 6 Performance analysis

This section evaluates the overhead cost of the TOD method. The evaluation is performed by using the following metrics, TagGenerationCost (the computational cost incurred in generation a tag), TagVerificationCost (the computational cost incurred in verifying a tag), and TagSize (the size of one tag). The last metric is for measuring storage cost at entities in the system.

In this evaluation, we assume that there are *K* data blocks in each data file, *DF*. After removing any redundant data block (as we only keep one copy of each data block, should there be multiple identical data blocks, the redundant or duplicated ones will be removed), the number of data blocks in a data file is reduced to *d* blocks. In each tag verification, the verifier requests *C* data blocks and the associated tags, which are randomly chosen from *d* data blocks, from the PCS provider.

When generating a tag, a number of operations are performed. The operations are for encryptions (symmetric and asymmetric), algebraic signature signing, BLS signature signing and modular additions in *GF*. These operations each consist of different types of basic operations, and each basic operation imposes a different level of computational cost. Table 7 lists the basic operations. The computational cost of each TOD operation is measured in terms of the numbers of different basic operations. Based on Eqs (3), (8), (9), (10), (12) and (11), we have TagGenerationCost for *d* without data blocks excluding the block encrytion opertions as: $d\times (2\times AS-G+Add_{AS}+2\times Exp_{Z_{n^{2}}}+Mult_{Z_{n^{2}}}+H1+H_{G_{1}}+Mult_{G_{1}}+2\times Exp_{G_{1}})$, where *d* is the number of data blocks in a file. When the data block encryption operations are included, TagGenerationCost is $d\times (2\times AS-G+Add_{AS}+Exp_{Z_{p}}+2\times Mult_{Z_{p}}+Add_{Z_{p}}+2\times Exp_{Z_{n^{2}}}+Mult_{Z_{n^{2}}}+H1+H_{G_{1}}+Mult_{G_{1}}+2\times Exp_{G_{1}})$. The computational complexity is O(*d*).

**Table 7:** Basic operations: Symbols and meanings.

Notation	Descriptions
$Mult_{G_{1}}$	Multiplication in *G*_1_
$EXP_{G_{1}}$	Exponentiation in *G*_1_
$Pair_{G_{1},G_{2}}$	Bilinear pairing e(x, y), x ∈ *G*_1_, y ∈ *G*_2_
*H*1	Cryptographic hashing, i.e. *H*1()
$H_{G_{1}}$	Hashing to *G*_1_ (i.e *H*())
*Add*_*Zp*_	Addition in Zp
*Mult*_*Zp*_	Multiplication in Zp
*Exp*_*Zp*_	Exponentiation in Zp
$Mult_{Z_{n^{2}}}$	Multiplication in *Z*_*n*^2^_
$Exp_{Z_{n^{2}}}$	Exponentiation in *Z*_*n*^2^_
*Add*_*AS*_	Addition in *GF*(2^*m*^)
*AS*-*G*	Cost of tag generation in AS

**Fig 12:**
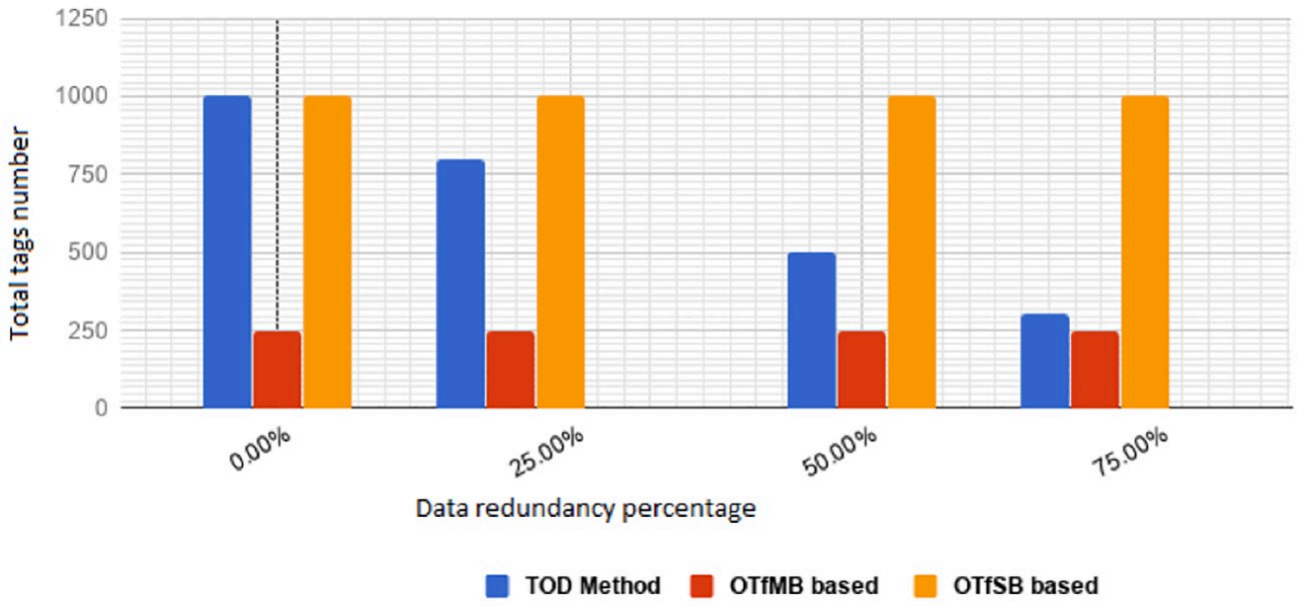
TOD method vs the OTfMB and OTfSB approaches: The number of tags generated against data redundancy percentage, *K* = 1000, total number of blocks in a data file, 4 Data Blocks are used in generating one tag in OTfMB approach.

The effect of tag deduplication on TagGenerationCost is captured by (*K* − *d*). In other words, the reduction in overhead cost as the result of tag deduplication is $\left(K-d\right)\times \left(2\times AS-G+Add_{AS}+2\times Exp_{Z_{n^{2}}}+Mult_{Z_{n^{2}}}+H1+H_{G_{1}}+Mult_{G_{1}}+2\times Exp_{G_{1}}\right)$ in the cases where encryption operations are included. Fig 12 illustrates overhead reductions due to the use of data deduplication in TOD by the total number of tags generated per file using our TOD method aginst two existing tag generation approaches, OTfMB and OTfSB. As shown in the figure, if there is no redundant data in a file, the total number of tags generated by the TOD method is identical to that by the OTfSB method. The more the redundant data it contains, the fewer the tags the TOD method generates. With the highest redundancy rate, the number of tags generated by the TOD method is closer to that with the OTfMB method. This indicates that TOD, by using data deduplication, can harvest the merits from both OTfSB and OTfMB. OTfSB offers a better level of security, in terms of unbounded verifiability, but produces more tags, whereas OTfMB produces less tags but is weak in assuring unbounded verifiability. TOD offers the same level of security, in terms of unbounded verifiability, as that by OTfSB but keeping the number of tags that need to be generated to the lowest level. It is worth mentioning that the cost saving by tag deduplication can also be applied across different files owned by the same PCS users. Excluding file ID and data block index number from tag generations allows us to achieve tag deduplication, which brings us the benefit of overhead cost reduction.

As both the algebraic signature and BLS signature algorithms have homomprphic property, the TOD method can perform both private and public tags verifications in an efficient and secure (without the need to access plaintext data) manner. A private tag verification only involves an algebraic signature generation cost and an addition operation to *GF* elements, which is considered fast and low cost, as it does not use any costly operations, such as modular exponentiation as in the case of RSA or pairing operations as in the case of BLS. According to EQ (9), EQ (16) and EQ (18), the computational cost incurred to a PCS user in verifying a single private tag, STagPriVerCost, is *AS*-*G* + *Add*_*AS*_ and the computational cost incurred to a PCS user in verifying *C* private tags, BTagPriVerCost, is *AS*-*G* + *C* × *Add*_*AS*_. The computational cost incurred to a TPA in verifying a single tag, STagPubVerCost, is $AS-G+2\times EXP_{Z_{n^{2}}}+2\times Mult_{Z_{n^{2}}}+H1+H_{G_{1}}+Exp_{G_{1}}+Mult_{G_{1}}+2\times Pair_{G_{1}G_{2}}$, and in verifying C public tags, BTagPubVerCost, is $AS-G+\left(C-1\right)\times Add_{AS}+\left(C+2\right)\times Mult_{Z_{n^{2}}}+4\times EXP_{Z_{n^{2}}}+C\times H1+C\times Multi_{G_{1}}+C\times H_{G_{1}}+Exp_{G_{1}}+\left(C-1\right)\times Add_{Z_{p}}+2\times Pair_{G_{1}G_{2}}$. So the computation complexity is O(C) for both the private verification and the public verification. Furthermore, applying the encryption operation for data confidentiality do not introduce any verification cost, as tags can be verified without decryption. Table 8 summarises the computational cost introduced to verifiers, i.e. the PCS user and TPA, in the TOD method. Table 9 compares TOD method with related tagging methods based on the specified requirements specified in Section 2.2.

**Table 8:** Tag generation and verification costs.

	PCS User	TPA
TagGenerationCost*	$K\times (2\times AS-G+Add_{AS}+2\times Exp_{Z_{n^{2}}}+Mult_{Z_{n^{2}}}+H1+H_{G_{1}}+Mult_{G_{1}}+2\times Exp_{G_{1}})$	-
TagGenerationCost**	$K\times (2\times AS-G+Add_{AS}+Exp_{Z_{p}}+2\times Mult_{Z_{p}}+Add_{Z_{p}}+2\times Exp_{Z_{n^{2}}}+Mult_{Z_{n^{2}}}+H1+H_{G_{1}}+Mult_{G_{1}}+2\times Exp_{G_{1}})$	-
STagPriVerCost	*AS-G* + *Add_AS_*	-
BTagPriVerCost	*AS*-*G* + *C* × *Add*_*AS*_	-
STagPubVerCost	-	$AS-G+2\times EXP_{Z_{n^{2}}}+2\times Mult_{Z_{n^{2}}}+H1+H_{G_{1}}+Exp_{G_{1}}+Mult_{G_{1}}+2\times Pair_{G_{1}G_{2}}$
BTagPubVerCost	-	$AS-G+C-1\times Add_{AS}+C+2\times Mult_{Z_{n^{2}}}+4\times EXP_{Z_{n^{2}}}+C\times H1+C\times Mult_{G_{1}}+C\times H_{G_{1}}+Exp_{G_{1}}+\left(C-1\right)\times Add_{Z_{p}}+2\times Pair_{G_{1}G_{2}}$

* Use non-encrypted data blocks,

** Use encrypted data blocks

**Table 9:** Comparing TOD method with existing tagging methods against the specified requirements.

Tagging Methods	F1	F2	S1	S2	S3	S4	S5	E1	E2		Cryptographic Schemes
								A PCS User	A PCS User + TPA		
Ateniese_1 method [3]*	Private	No	Yes	Yes	No	No	No	O(T×NT)	O(1)/O(NT)	-	HF+SC
Chen method [4]*	Private	No	Yes	Yes***	No	No	No	O(T×NT)	O(1)	-	AS+SC
Krishra method [5]	Private	No	Yes	No	No	No	No	O(K)	O(1)	-	SC
Luo_1 method [6]**	Private	No	Yes	No	No	No	Yes	O(K)	O(C)	-	AS
Sookhak method [7]**	Private	No	No	No	No	No	Yes	O(K×S)	O(1)	-	AS
Zhang method [18]**	Private	No	Yes	Yes	No	No	Yes	O(K)	O(C)	-	MAC
Xu method [19]**	Private	No	Yes	Yes	No	No	Yes	O(K×S)	O(1)	-	HomMAC
Ateniese_2 method [8]**	Public	No	Yes	Yes	Yes	Yes	Yes	O(K)	-	O(1)	RSA
Ni method [9] **	Public	No	Yes	Yes	Yes	No	Yes	O(K×S)	-	O(1)	RSA
Erway method [23] **	Public	No	Yes	Yes	Yes	Yes	Yes	O(K×S)	-	O(1)	RSA
Hanser method [10]**	Public	No	Yes	Yes	Yes	Yes	Yes	O(K)	-	O(1)	ECDSA
Li method [11]**	Public	No	Yes	Yes	Yes	No	Yes	O(K×S)	-	O(C)	BLS
Liu method [12]**	Public	Yes	Yes	Yes	Yes	No	Yes	O(K×S)	-	O(C)	BLS
Wang method [17, 30, 31]**	Public	No	Yes	Yes	Yes	No	Yes	O(K)	-	O(C)	BLS
Yang method [32]**	Public	Yes	Yes	Yes	Yes	No	Yes	O(K×S)	-	O(C)	BLS
Luo_2 method [13]**	Public	Yes	Yes	Yes***	Yes	No	Yes	O(K×S)	-	O(C)	BLS
Salim method [13]**	Public	Yes	Yes	Yes***	Yes	No	Yes	O(K)	-	O(C)	BLS
TOD method	Both	Yes	Yes	Yes	Yes	Yes	Yes	O(d)	O(C)	O(C)	HE + AS + BLS

* OTfMB approach,

** OTfSB approach approach, + in the case of private variability,

*** only the collision between data blocks of one User, SC is Symmetric Cipher, HF is Hash Function, AS is Algebraic Signature, HE is Homomorphic Encryption.

**Fig 13:**
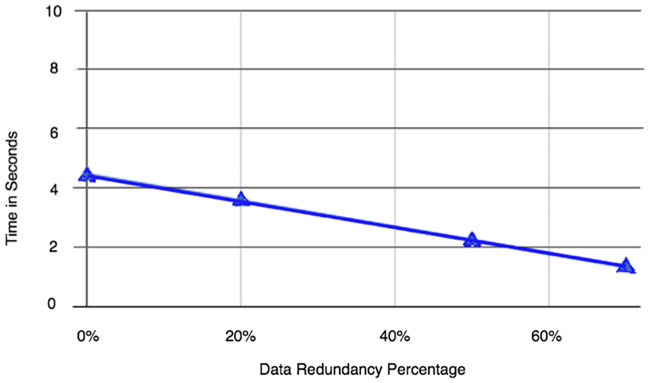
The required time of encryption vs. data redundancy percentage.

Addition to the above theoretical analysis, we have also carried out experiments to evaluate the computational costs of the TOD method further and compared the costs with those of related tagging methods. For this, we have produced a prototype of the TOD method using Java. The experiment is run on a system with Intel Core i5 at 2.4 GHz and 4GB RAM. For implementing cryptographic primitives required in the TOD, e.g. a secure random number generator, a hash function (e.g. SHA3-384), and digital signatures (e.g. RSA and BLS), Java Cryptography Extension (JCE) [48] and Java Pairing-Based Cryptography (JPBC) [49] are used. The data block size used is 25 kilobytes (KB). We have evaluated the benefit brought by data deduplication by measuring the times required for encrypting 1000 data blocks, i.e. *K* = 1000, versus different levels of data redundancy. Fig 13 shows the effect of data deduplication on reducing the encryption time under different data redundant percentages.

**Fig 14:**
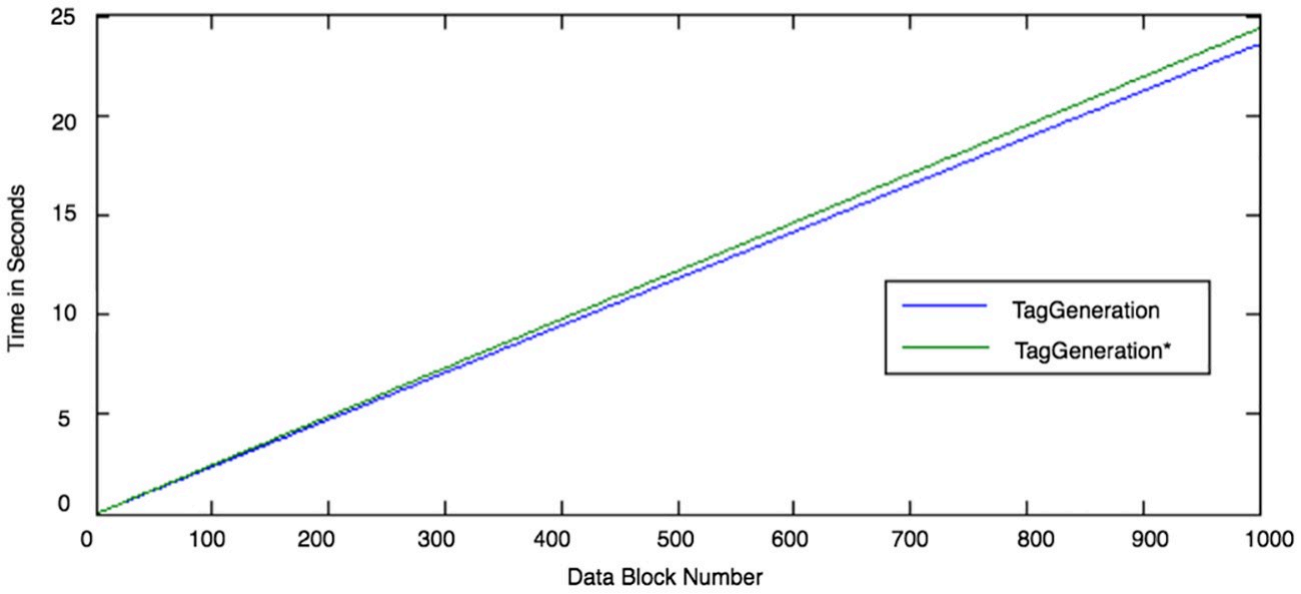
Tag generation cost vs data blocks number.

We have evaluated the tag generation times for a single tag and for a whole file (consisted of 1000 data blocks) and compared the results from the TOD method with those from the related tag generation methods. The results are shown in Table 10. From the table, it can be seen that among the eight tag generation methods, the RSA based method takes the longest time, so it is the most expensive method. The cheapest methods are those symmetric key based, e.g. MAC and AS based methods. However, these methods do not provide non-repudiation service. In comparison with other public key based methods, such as the BLS based, the TOD method is more expensive, 2.7 times higher. This is the price for providing enhanced functionality, as TOD offers both public and private verifiability. Furthermore, Fig 14 compares the tag generation times with and without encrypting the data blocks. The results show that the additional cost introduced by the encryption operation is negligible.

**Table 10:** Comparing the TOD method and the related works: The required time of tag generation (in seconds).

Methods	One Tag	1000 Tags
Hash based	0.0495	12.6741
AS based *	0.0413	10.5789
AS based**	0.0021	2.0466
MAC based	0.0096	9.5461
RSA based	0.4080	408.0272
ECDSA based	0.0355	35.4468
BLS based	0.0075	7.5000
TOD	0.0280	28

* OTfMB approach,

** OTfSB approach

Table 11 compared the TOD method with the BLS based method in terms of tag verification times. From the figure, it can be seen that the times taken about twice as much as what is taken by the BLS method for public verifications, but only 0.39% of the time taken by the BLS method for private verifications.

**Table 11:** TOD method vs. BLS based tagging method: The required time (in seconds) of private and public tag verification.

	TOD	BLS-based
Public Verification	0.0514	0.0259
Private Verification	0.0001	0.0259

**Fig 15:**
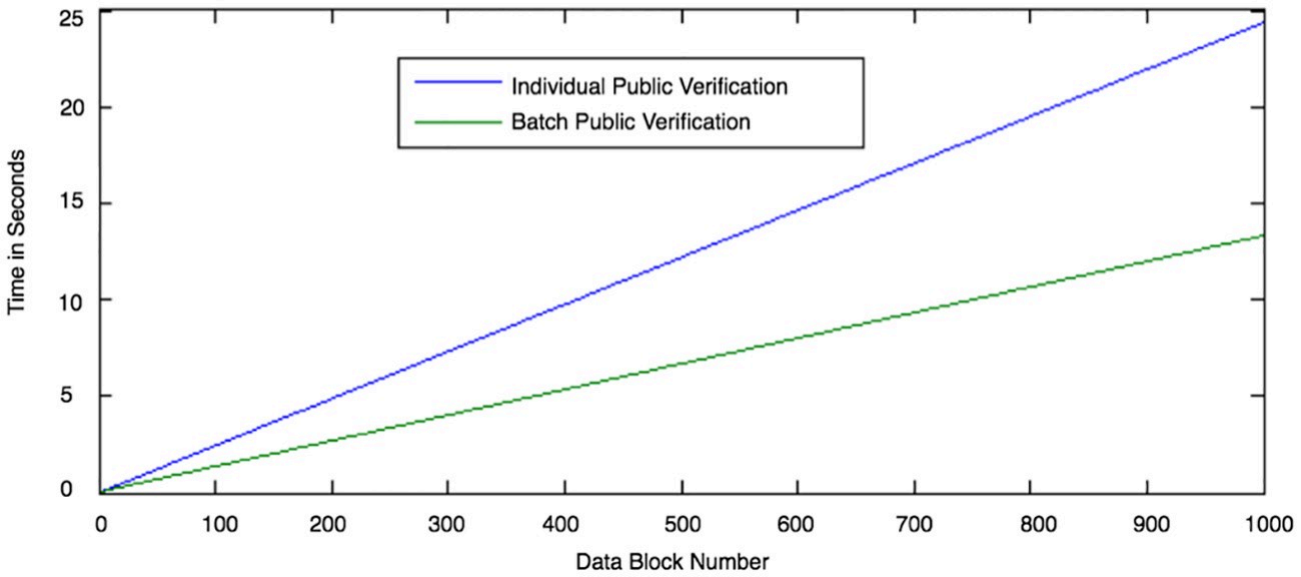
Public tag verification cost: Individual vs batch verifications.

**Fig 16:**
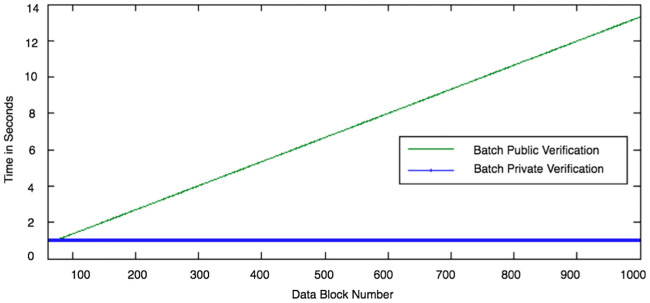
Batch verification cost: Private vs public.

Fig 15 compares the batch tag public verification time with single tag public verification time. From the figure it can be seen that, by using batch verifications, the time taken in verifying the integrity of a file can be reduced significantly; the more the data blocks a file consisted of, the higher the reduction. For example, for a file consisted of 1000 data blocks, the verification time is reduced by nearly 45% when batch verification is used. Fig 16 compares batch tag private verification time with that of batch tag public verifications. The results show that batch tag private verification time is virtually independent of the number of tags involved, whereas batch tag public verification time increases linearly as the number of tags involved increases, and the former is only a fraction of the latter.

The above results are significant. It indicates that, in supporting both public and private verifiability, TOD mainly introduces additional cost to the TPA. The integrated support of private verifiability, which allows PCS users to monitor the integrity of their service provider, only imposes a negligible level of verification cost on the PCS users. In other words, TOD does not require that PCS users should trust the service providers and additional cost introduced to the users as the result of having this feature is negligible.

The sizes of tags influence security levels as well as storage and communication costs. In TOD, each data block is tagged with five tags, i.e. *IDTag*_*i*_, *En*_*IDTag*_*i*_, *DBTag*_*i*_ and *DBTagTag*_*i*_, where {*IDTag*_*i*_}, {*DBTag*_*i*_} are used for private verifications and {*En*_*IDTag*_*i*_}, {*DBTag*_*i*_} and {*DBTagTag*_*i*_} are used for public verifications. *IDTag*_*i*_, and *DBTag*_*i*_ are generated using the AS scheme, using *GF*(2^*m*^), meaning that each tag size is m-bits long. *En*_*IDTag*_*i*_ is encrypted *IDTag*. *DBTagTag*_*i*_ is generated using the BLS signature, and its length varies with the security level the signature provides. As discussed in Section 5, the sizes of these tags should also take into account of forgery and collision resistance levels. Based on the security analyisis, to ensure a strong level of collision resistance, both tags (i.e each AS tag and BLS tags) are assumed to have the length of 32 bytes long (i.e. *m* = 256 bits and $L_{E_{G_{1}}}=256$ bits). {*IDTag*_*i*_} are stored locally at the user-side and {*En*_*IDTag*_*i*_} with TPA, thus, the total size of a tag that is stored at the PCS server is *DBTag*_*i*_ + *DBTagTag*_*i*_ = 32 bytes + 32 bytes = 64 bytes. Table 12 compares the tag size in bytes of the TOD method with those of existing methods. In this comparison, the SHA3-384 (i.e. 48 bytes) is used as the underlying hash function for MAC-based tags. As shown in the table, algebraic signature and BLS based methods generate the shortest tag size, whereas RSA based method generates the longest. The tag size of our TOD method is 64 bytes which is higher than the tag sizes produced by the algebraic signature based and BLS based methods. However, different from the algebraic signature and BLS based methods, which only supports private verifiability and public verifiability, receptively, the TOD method supports integrated public and private verifiability. The storage cost complexities of the user and TPA are based on whether *IDTags* and *En*_*IDTags* are kept by the user and TPA, respectively, in DIA, or not. If yes, the storage cost complexity is O(d) at both the user and TPA.

**Table 12:** TOD method vs the related work: The tag size generated (in bytes).

Works	Tag Size
Hash function-based	48
Algebraic Signature based *	32
Algebraic Signature based **	32
MAC-based	48
RSA-based	384
ECDSA-based	64
BLS-based	32
TOD Method	64

* is *OTfMB approach*,

** is *OTfSB approach*

## 7 Conclusion

This paper has proposed and evaluated a novel method, called Tagging of Outsourced Data (TOD), that can be used in DIA to address the issue of how to check the integrity of data that are managed by third parties periodically without downloading the whole data from PCS. The paper has also presented a comprehensive security analysis and theoretical and experimental evaluation of the overhead costs of the method. The evaluation results are compared with those of related tagging methods. The analysis and comparison results indicate that, in comparison with related methods, TOD is more efficient, particularly for the user ends, and provides richer functionality, including providing a stronger level of security protections to data.

Our future work includes the design of DIA framework that employs the TOD method. As TOD method is based on spot-checking, like the existing methods, it should emphasise there is a required number of picked data blocks for verification, i.e., C, to detect misbehaviour of the PCS provider with high probabilities. Thus, it can use nonces as the second layer to prevent replay attacks. Furthermore, the design of a data structure to support dynamic data and data deduplication among the user’s data file in PCS. Finally, we can try to find a solution to release the PCS user from keeping *IDTags* locally with the DIA design.
